# Evaluation of the effect of dietary supplementation with *Allium mongolicum* regel bulb powder on the volatile compound and lipid profiles of the *longissimus thoracis* in Angus calves based on GC–IMS and lipidomic analysis

**DOI:** 10.1016/j.fochx.2024.101820

**Published:** 2024-09-10

**Authors:** Wangjing Liu, Huixia Gao, Jianjian He, Aihuan Yu, Chenxu Sun, Yaodi Xie, Haibo Yao, He Wang, Yueyan Duan, Jinsheng Hu, Zhaomin Lei, Defu Tang

**Affiliations:** a College of Animal Science and Technology, Gansu Agricultural University, No. 1 Yingmen Village Anning, Lanzhou, Gansu, 730070, People's Republic of China; b Tianjin Halo Biotechnology Co., Ltd., No. 18 Gui Yuan Road, Huan Yuan Hi Tech-Industrial Area, Tianjin, 300384, People's Republic of China

**Keywords:** Flavonoids, Polyphenolic compounds, Meat flavour, GC–IMS, Lipidomics

## Abstract

The effect of *A. mongolicum* Regel bulb powder (AMRP) supplementation on the flavour of beef from Angus calves has not been investigated thus far. We used GC–IMS and untargeted lipidomics techniques to examine the volatile compound and lipid metabolic profiles and reveal the effects of dietary AMRP supplementation on the flavour of beef. A total of 6 characteristic volatile compounds and 30 key lipid compounds were identified in the AMRP treatment group. AMRP promoted the release of triglycerides and phosphatidylinositols from beef and accelerated the production of volatile compounds such as ethyl acetate, 1-penten-3-one, and tetrahydrofurane, and the production of these three characteristic volatile compounds was significantly correlated with the UFAs in triglycerides according to correlation analysis. In summary, dietary AMRP supplementation had a positive effect on the flavour of beef, and these findings provide a theoretical basis for the development and utilisation of AMRP as a feed additive.

## Highlights

• The role of AMRP in the volatile compound and lipid profiles of beef was reported for the first time.
• 27 volatile compounds were detected in *Allium mongolicum* Regel bulb using GC–IMS.
• 47 volatile compounds were detected in raw beef using GC–IMS.
• 199 differential metabolites were identified, 112 were upregulated and 87 were downregulated.
• The generation of characteristic volatile compounds is related to the UFAs profile.

## 1 Introduction

Historically, consumer acceptance of beef has depended on its tenderness, but with the development of genetics, breeding disciplines, and guidance from ranch managers, beef tenderness has improved (Gonzalez, 2022). However, recent studies have shown that consumers are increasingly choosing flavour as the most critical attribute of beef, surpassing the importance of meat colour as well as freshness (Chail et al., 2016, Chail et al., 2017). Kerth et al. (2024) suggested that flavour encompasses the impact of the combination of taste, aroma and intraoral sensations. Among the descriptive attributes of beef, positive (beef fat-like, beef identity, and roasted, umami, sweet) and negative (mouldy, cardboard, and sour aromas) characteristics are closely related to consumer preference attributes (Miller et al., 2023). When identifying drivers of consumer flavour preferences for beef, we often overlook the fact that different consumers have different preferences of the degree of doneness of beef. It is well known that the lipid oxidation that occurs in beef during the cooking process and the accompanying Maillard reaction are the main factors contributing to the formation of beef flavour (Kerth & Miller, 2015). When beef is overcooked, cardboardy and liver-like flavours resulting from dramatically increased levels of lipid oxidation are flavour attributes that most consumers dislike (Miller et al., 2023). However, beef that is raw, hardly cooked or half cooked has a greater amount of blood than fully cooked beef, resulting in a slightly sweet aroma and a metallic flavour (Miller et al., 2022). Both the odour of raw meat and the cooked meat are invariably related to the presence of non-volatile precursors in raw meat due to lipid oxidation, and these compounds are responsible for the development of beef flavour (Resconi et al., 2018). There has been a great deal of previous research on beef flavour after cooking (Jiang et al., 2024; Watanabe et al., 2015; Zou et al., 2018), and numerous compounds that are already present or produced in raw meat are still present after cooking and may affect flavour perception; however, there is relatively few studies on the generation and evolutionary pathways of the flavour of raw beef (Saraiva et al., 2015). Although the odour of raw meat becomes less important compared to its colour and appearance when consumers purchase beef in supermarkets or other places, as most beef is packaged, we need to ensure that the odour is also acceptable when consumers open the packaging (Schindler et al., 2010).

The most important source of volatile compounds in raw meat is the lipid fraction (Xiong et al., 2022), mainly including phospholipids, which produce many volatile compounds such as acids, aldehydes, ketones, and alcohols through the phenomenon of auto-oxidation and the abovementioned biochemical processes that take place even under refrigeration, vacuum, and dark conditions (Ai-Dalali et al., 2022). Wu and Wang (2019) reported that different lipid compositions [e.g., phosphatidylcholine (PC), phosphatidylethanolamine (PE), and lipids such as triglyceride (TG), diglyceride (DG), and phosphatidylinositol (PI)] may affect the formation of volatile compounds during lipid oxidation, while *s*n-1 site saturated fatty acids (SFAs) and *s*n-2 site unsaturated fatty acids (UFAs) may also have an effect on the thermo-oxidative stability of lipids. In addition to lipids, precursors such as phosphorylated monosaccharides, amino acids and ribose in raw meat are equally important for flavour formation in both raw and cooked meat (Zou et al., 2019). These precursors in raw meat are influenced by feeding regime, diet and additives, postslaughter ageing and their interactions (Khan et al., 2015), where natural plant-derived additives rich in phenolic compounds are able to influence the production of these flavour precursors in beef and retard lipid oxidation to improve beef palatability (Ponnampalam et al., 2022). He et al. (2023) reported that the addition of 260 mg/d/calf oregano extract (main components: thymol and carvacrol) to the diet increased polyunsaturated fatty acids (PUFAs) and conjugated linoleic acid content in the muscle of Pingliang red cattle by modulating rumen biohydrogenation and reducing SFA, and additionally increased flavour amino acids by decreasing protein degradation in the rumen and allowing flavour amino acids to enter the small intestine. Flavour-producing amino acids such as lysine, glutamic acid and aspartic acid in the muscle to improve beef flavour. De Zawadzki et al. (2017) reported that the addition of mate (*Ilex paraguariensis A.St.-Hil.*) extract (main components: alkaloids, saponins, and phenolic acids) to Nellore steer diets to increase the content of umami substances (inosine and carnosine) in fresh beef meat was preferred by consumers. *Allium mongolicum* Regel belongs to the genus *Allium* and has been widely reported to have beneficial effects on metabolic syndrome (obesity, dyslipidaemia, hypertension, and type II diabetes mellitus), mainly due to its high content of flavonoids, steroidal saponins, stilbenoids and organosulfur compounds (Taleghani et al., 2024). Our previous study reported that *A. mongolicum* Regel powder (AMRP) and its extracts (main components: flavonoids and polyphenolic compounds) were able to significantly improve the intramuscular fatty acid (FA) composition of lamb and reduce the content of branched-chain FAs, such as 4-methyloctanoic acid, 4-methylnonanoic acid, and 4-ethyl-octanoic acid, which are associated with the distinctive flavour and odour of lamb meat, thus improving the flavour (Liu et al., 2022). The ability of phenolic compounds in AMRP to induce differential muscle and fat epigenetic regulation and reduce transcriptomic differences between tissues highlights its biological function, revealing a regulatory mechanism for improving intramuscular fat (IMF) deposition in sheep as well as lamb odour (Xue et al., 2021). Moreover, Fan et al. (2023) identified differential miRNA and mRNA candidates related to lipid metabolism in adipose tissue by transcriptome sequencing technology and found differential miRNAs and mRNAs involved in the lipogenesis, FA oxidation, and FA transport processes, which provided a plausible explanation for the modulation of FA composition and flavour of mutton by AMRP supplementation. In summary, the improvement in the flavour of animal meat products due to dietary AMRP supplementation is related to its ability to modulate FA synthesis and lipid metabolite production, but to our knowledge, there is no literature on the effect of AMRP supplementation on the flavour or lipid composition of beef or on the mechanism by which these two parameters interact.

The gas chromatography tandem ion mobility spectrometry (GC–IMS) flavour analysis technique is based on the excellent separation capability of GC combined with the advantages of the high sensitivity and fast response of IMS to highly electronegative or high-proton affinity groups, such as amino groups, hydrophobic groups, aldehydes, ketones, and aromatic compounds in flavouring substances of meat and meat products, and improves the accuracy of the analysis of characteristic volatile compounds; thus, this technique is widely applicable and yields remarkable results (Li et al., 2019; Wan et al., 2023; Zheng et al., 2024). We recently reported that the addition of 20 g/d/calf of AMRP to the diet significantly increased monounsaturated fatty acid content in the *longissimus thoracis* (LT) and improved meat quality (Liu et al., 2024). Meat flavour generation is related to lipid composition, and lipidomics enables the comparison of lipid compound species and lipid metabolism between treatment groups and the identification of key lipid metabolites, ultimately revealing the roles and formation mechanisms of lipid metabolites in beef flavour (Xiong et al., 2022). Muscle lipidomic research methods can comprehensively reveal the intramuscular lipid profile of Angus beef, which is important for understanding and screening lipid molecules associated with optimal beef flavour.

We have reported in our recent studies that supplementation of 10 g of AMRP per Angus calf per day significantly increased their daily weight gain and feed efficiency, and supplementation of 20 g of AMRP per Angus calf per day significantly improved beef tenderness, water retention, and increased antioxidant properties and UFAs (Liu et al., 2024). This study was the first to investigate the effects of dietary AMRP supplementation on the volatile compound and lipid profiles of the *longissimus thoracis* in Angus calves. First, GC–IMS and liquid chromatography–mass spectrometry (LC–MS/MS) nontargeted lipidomic techniques were used to detect volatile and lipid metabolism profiles and to reveal the changes in volatile and lipid profiles of Angus calves caused by dietary AMRP supplementation. The correlation between key lipid metabolites and characteristic volatile flavour compounds was further analysed, and key lipid compounds affecting beef flavour were screened. This study will have a positive impact on the development of beef cattle industrialisation and beef flavour improvement.

## 2 Materials and methods

### 2.1 Preparation of AMRP, Allium mongolicum regel flower powder, and Allium mongolicum regel root powder

Fresh *A. mongolicum* Regel was collected during the blooming stage in August 2022 in the Minqin area (situated at 39°27′ N, 101°49′ E), Gansu, China. Throughout August, we collected intact *A. mongolicum* Regel plants in five batches, stored them at 4 °C, and transported them to the laboratory for accurate separation of roots, bulbs, and flowers for subsequent processing. In the laboratory, the cleaned bulbs, flowers and roots were stored flat in a large hot-air drying oven (DHG-9035 A, manufacturer: Kexiao Scientific Instrument Co., Ltd., Shanghai, China). The drying temperature was set below 65 °C to avoid the loss of active ingredients. Thereafter, the dried bulbs, flowers and roots were crushed into powder using a herbal medicine crusher (XL-60C, manufacturer: Xulang Machinery Equipment Co., Ltd. Guangzhou, China), and the resulting material was passed through a 1 mm sieve. Adequate amounts of *A. mongolicum* Regel bulb powder (AMRP), derived from a mix of five batches of AMRP, were used in animal feeding tests. The AMRP and the *A. mongolicum* Regel flower and *A. mongolicum* Regel root powders were used for volatile compound detection. All measurements were carried out in five replicates (from five batches of AMRP).

### 2.2 Animals and diets

This study was approved by the Animal Ethics Committee of Gansu Agriculture University, Lanzhou, China (approval number: 2006–398). The study was conducted between April and August 2023 at Longshengda Livestock Breeding Co., Tianjin, China (39°95′ N, 116°52′ E). Twenty-four healthy black Angus heifers (average body weight = 280.4 ± 15.74 kg, average age = 12 ± 0.3 months) of the same genetic background (Timaru, New Zealand) were randomly assigned to four feed groups: the control (C) group; the basal diet without any supplementation group; and the L, M, and H groups with the basal diet supplemented with 10, 15, and 20 g of AMRP per calf per day, respectively. One hundred grams of a concentrate of the basal diet was mixed with 10, 15, and 20 g of AMRP respectively, and each mixture was divided into two equal amounts and then they were provided for individual calf twice daily (at 0800 h and 1730 h) to ensure that the supplements were completely consumed by each calf. Each treatment group was composed of three pens, with two calves in each pen (each pen = 3 × 6 m). Each pen equipped with a feed trough and water trough for feeding and free access to fresh water. The basal diet was formulated to satisfy the Nutrient Requirements of Beef Cattle by the National Academies of Sciences, Engineering, and Medicine (NASEM) (2016). The dietary ingredients and nutrient levels of the basal diet are shown in Supplemental Table 1. Nutrients contents and fatty acid composition of AMRP are shown in Supplemental Table 2. Analysis conventional nutrient and FA composition of the basal diet and AMRP has been reported in Dugan et al. (2007).

### 2.3 Muscle sampling

The pre-experiment lasted 15 days, and the fattening period lasted 120 days. The Angus calves were slaughtered with an average final body weight of 390.04 ± 12.85 kg. Subsequently, the calves were transported from the fattening farm to the slaughterhouse (Tianjin Jinyifeng Foodstuffs Co., Ltd., 39°22′ N, 117°14′ E) by a medium-sized lorry for slaughter, with a transport distance of 75.1 km and a transport time of 2 h. Four groups of cattle were slaughtered on each of the four days spanning August 20–23 using a halal slaughtering practice. The slaughtering process was carried out in a strictly traditional halal manner, and the left *longissimus thoracis* (LT) was collected at the 12th rib of the carcass, packaged in a polystyrene vacuum packing bag (17 cm × 25 cm), and stored at −80 °C for volatile compound and lipidomic testing. The specific process of Halal slaughtering is as follows: Halal slaughtering requires the slaughtering of live animals: during halal slaughtering, it must be operated by a qualified imam. This type of slaughter does not allow the use of electric hemp or other mechanical devices to render the animal unconscious, but must be carried out directly by a human being. Before slaughtering, the operator should face the direction of Mecca and recite the “In the name of Allah” as a start-up prayer for slaughtering. Special knife techniques and blood handling: Islam requires that animals be slaughtered with a swift and precise knife, that the blade be sharp, that the throat be cut, and that the four tubes (arteries, veins, oesophagus, and trachea) be severed in a single cut to ensure that the blood flows out completely. Halal slaughtering must be carried out by the imam himself, which reflects the strict requirements of the religion on the slaughtering process.

### 2.4 Volatile compound analysis in the roots, bulbs, and flowers of *A. mongolicum* regel and in LT samples determined by GC–IMS

Volatile compounds were detected in AMRP samples, *A. mongolicum* Regel root and flower samples, and LT samples from the C, L, M, and H groups using a GC–IMS flavour analyser (FlavourSpec®, G.A.S., Dortmund, Germany) according to the methods of Li et al. (2019) with slight modifications. Two grams of the sample to be measured was placed into a 20 mL headspace injection vial and sealed. After incubation at 60 °C for 15 min, 500 μL of headspace sample was automatically injected into the syringe via an 85 °C injection needle at an incubation speed of 500 rpm. Subsequently, the sample was fed into the FS-SE-54-CB-1 capillary column (15 m × 0.53 mm) with high-purity nitrogen (≥ 99.999 %) at a starting flow rate of 2 mL/min, and the procedure was as follows: 2 mL/min for 2 min, 100 mL/min for 8 min, and 100 mL/min for 10 min. After isothermal elution at 60 °C, the sample underwent primary separation on a gas chromatographic column and subsequently entered the ion mobility tube. The tested molecule is ionized in the ionisation zone for secondary separation. They were then introduced into the drift tube, which was operated at a temperature of 45 °C and a drift gas (nitrogen) flow rate of 150 mL/min. Every spectrum was recorded with an average of 12 scans. The GC–IMS system was operated with VOCal software for viewing analytical spectra and qualitative and quantitative data, and the application's built-in NIST and IMS databases were applied for qualitative analysis of analytes. The Reporter plug-in was used to directly compare spectral differences (three-dimensional, two-dimensional topographic plots and two-dimensional differential topographic plots between different samples). The Gallery Plot plug-in was used for the construction of volatile compound fingerprints to compare differences in volatile compounds between samples visually and quantitatively. The data are the means from five biological replicates.

### 2.5 Lipidomic analysis of LT samples

#### 2.5.1 Sample pretreatment

For sample preparation and lipid extraction, lipids were extracted according to MTBE method to ensure comprehensive coverage of lipid classes and subclasses (Matyash et al., 2008). For the lipid extraction process, 20 mg of lyophilised LT sample was taken, weighed accurately, added to a precooled 500 μL 2:2:1 methanol/acetonitrile/water (*v*/v/v) mixture, vortexed and mixed for 30 s, sonicated for 30 min and then left to stand for 10 min at −20 °C. The organic phase was separated by centrifugation at 4 °C for 20 min at 14,000 ×*g*. The organic phase was then separated by centrifugation at 4 °C for 20 min. The supernatant containing 400 μL of lipid extract was transferred to an EP vial and dried in a vacuum concentrator (IKA HB10, IKA India Private Limited, Germany). Then, the lipid extracts were redissolved in 100 μL of 1:1 acetonitrile/water (*v*/v) solution, vortexed at 4 °C for 2 min, sonicated for 5 min, and centrifuged at 14,000 ×*g* for 20 min before separation. Finally, 100 μL of the supernatant was carefully transferred to a sample vial for analysis.

#### 2.5.2 LC–MS/MS analysis

Lipid separation was performed on an Agilent 1290 Infinity LC UHPLC system equipped with an ACQUITY UPLC BEH Amide chromatographic column (1.5 μm, 2.1 mm × 100 mm, Agilent, California, USA) with a column temperature of 25 °C, a flow rate of 0.5 mL/min and an injection volume of 2 μL. The mobile phase consisted of water +25 mM ammonium acetate +25 mM ammonia (A) and acetonitrile (B). The elution gradient of the mobile phase was as follows: 0–0.5 min, 5 %/95 % A/B; 0.5–7 min, 35 %/65 % A/B; 7–8 min, 50 %/50 % A/B; 8–9 min, 60 %/40 % A/B; 9–9.1 min, 80 %/20 % A/B; and 9.1–12 min, 100 %/0 % A/B. The samples were placed in a 4 °C autosampler throughout the analysis. To avoid the effects caused by fluctuations in the instrumental detection signal, the samples were analysed continuously in a random order. QC samples were inserted into the sample queue for monitoring and evaluating the stability of the system and the reliability of the experimental data.

The retention index (RI) of each compound was calculated using ketone C4-C9 (Sinopharm Chemical Reagent Beijing Co., Ltd.) as an external reference. MS analyses were performed using an AB Triple TOF 6600 mass spectrometer (AB SCIEX, Massachusetts, USA), and the primary and secondary spectra of the samples were acquired in positive and negative ionisation modes. The parameters were set as follows: collision energy (CE), 35 V ± 15 eV; declustering potential (DP), 60 V (+) and − 60 V (−); isotopes within 4 Da were excluded; and the number of candidate ions for monitoring each cycle, 10. Lipids were identified by matching precursors and characteristic fragment masses using Lipidsearch (Version 4.2, Thermo Fisher Scientific, Waltham, MA, USA). Lipid species were identified using the LipidSearch software to process the raw data and for peak alignment, retention time correction and extraction peak area. LipidSearch contains data on more than 30 lipid classes, with information on more than 1,700,000 ion fragments. Adducts of ^+^H, ^+^NH_4_ were selected for positive mode searches, and ^—^H, ^+^CH_3_COO were selected for negative mode searches since ammonium acetate was used in the mobile phases.For the data extracted from LipidSearch, remove the ion peak with a value of>50 % missing from the group. Normalization and integration using the Perato scaling method. The relative quantifications of lipids were calculated by comparing their relative peak areas. The data are the means from six biological replicates.

### 2.6 Statistical analysis

Statistical analyses were performed using the GLM procedure in SPSS (version 22.0; SPSS, Chicago, IL, USA) for ANOVA. The analytical model for volatile compounds in *A. mongolicum* Regel root, bulb and flower powder samples included fixed effects for the different part (root, bulb and flower) treatments, as well as 5 replicates for each treatment, which we consider as random effects. The analytical model for volatile compounds in LT samples included fixed effects for treatments of the different diets (groups C, L, M, and H), as well as treating each pen as a random effect. Fixed effects for treatments of different diets (groups C and H) were included in the analytical model for lipid compounds, treating each pen as a random effect. *P* < 0.05 in Duncan's test of significant difference was considered to indicate a significant difference, and *P* < 0.01 was considered to indicate a highly significant difference. Relative lipid concentrations were normalized to the protein concentration for each sample, and the normalized concentrations were then used for analysis. Resulting data files were imported into MetaboAnalyst to perform the multivariate analysis. The characteristic flavour compounds of LT in the C and H groups were visualized using the GraphPad Prism 9.5 package (GraphPad Inc., La Jolla, CA, USA). To evaluate the correlation between the key lipid compounds and the characteristic volatile compounds, the correlation coefficients were calculated using cor.mtest () in R. A *P* value of less than 0.05 was considered significant, and the correlation plots were plotted by the corrplot software package for R. We referred to the data analysis method of Biffin et al. (2020).

## 3 Results and discussion

### 3.1 Screening of characteristic volatile compounds in AMRP samples

We constructed fingerprints of volatile compounds from different parts of *A. mongolicum* Regel (roots, bulbs and flowers) by GC–IMS (Supplemental Fig. 1 A). The 27 volatile compounds in the AMRP samples were qualitatively analysed based on the RTs and ion migration times of the volatile substances (Supplemental Table 3). These volatile compounds mainly included 7 esters, 6 alcohols, 5 heterocycles, 3 ketones, 2 aldehydes, and 4 others. The PCA model revealed discrete differences in volatile compound data among samples from different parts of *A. mongolicum* Regel, with significant separation of volatile compounds in *A. mongolicum* Regel bulb, flower, and root powders (Supplemental Fig. 1 B), suggesting that there are significant differences in flavour between different components of *A. mongolicum* Regel. As shown in Supplemental Fig. 1C and Supplemental Table 4, a total of 12 characteristic volatile flavour compounds were selected based on the screening conditions of a variable importance in the projection (VIP) ≥ 1 and *P* < 0.05. Among the detected compounds, 1-pentanol, isobutyl butyrate, isopentyl formate, and acetophenone were highly significantly more abundant in AMRP than in the flowers and roots (*P* < 0.01).

### 3.2 Volatile compound analysis of LT by GC–IMS

#### 3.2.1 Visual topographic plots

**Fig. 1:**
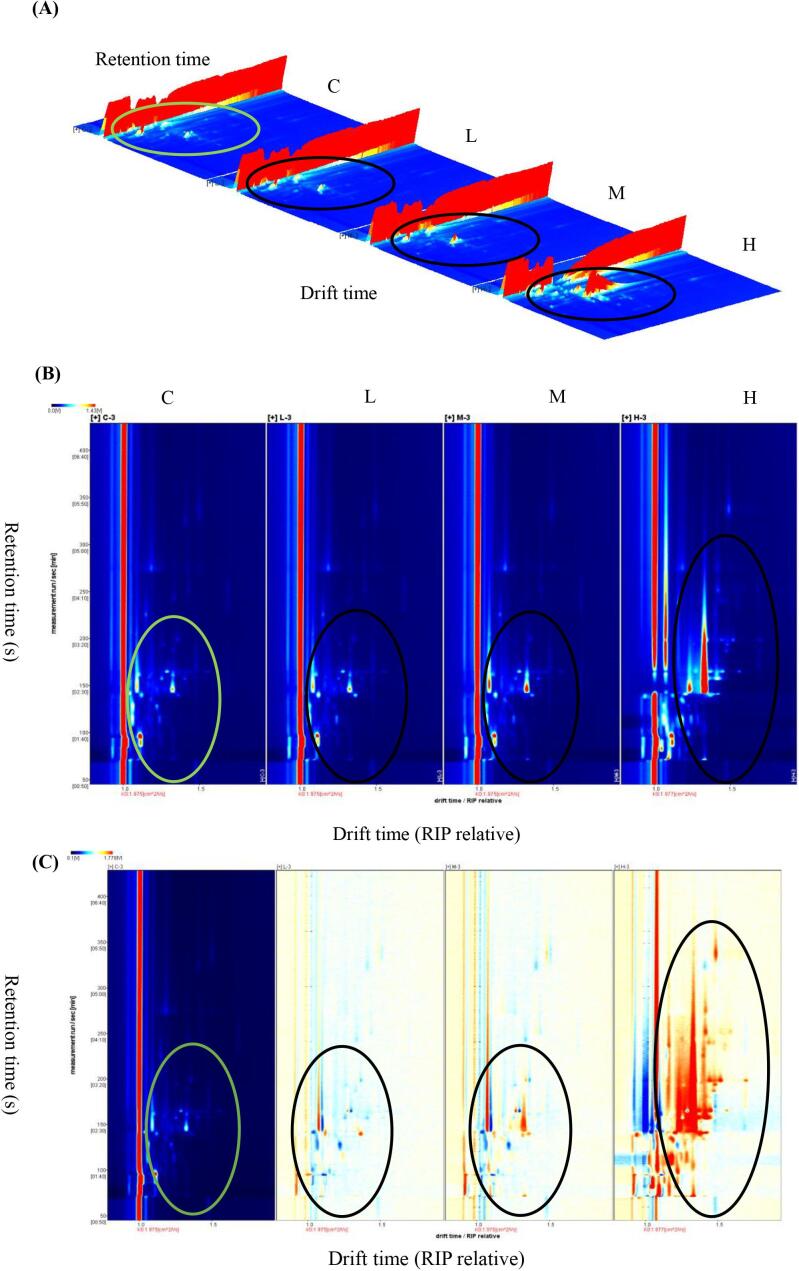
Three-dimensional (A) and two-dimensional topographic plots (B) and two-dimensional differential topographic plots (C) of volatile compounds in the basal diet without additives and after dietary *Allium mongolicum* Regel powder (AMRP) supplementation in the *longissimus thoracis* of Angus calves. C, a basal diet without additives; L, M and H indicate 10, 15 or 20 g per Angus calf per day of AMRP supplementation in the basal diet, respectively. The number of observations for each mean value was three (*n* = 5).

We analysed volatile compounds in beef from Group C and the *A. mongolicum* Regel-treated groups (L, M and H groups) by GC–IMS, and the data were visually represented using three-dimensional topographical maps (Fig. 1A) to assess the differences in flavour profiles. As the level of AMRP addition increased, the variety of volatile compounds in the beef increased, and the peak signal intensities increased significantly. The results of our previous study confirmed that the addition of different levels of AMRP and its extracts to the diet reduced the concentrations of 4-methyloctanoic acid and 4-ethyloctanoic acid in the perirenal adipose tissue, dorsal subcutaneous adipose tissue, and omental adipose tissue of small-tailed Han sheep and that these branched-chain FAs were significantly correlated with the characteristic flavour and odour of lamb meat, particularly the unpleasant odour of mutton (Liu & Ao, 2021). The rate of action of AMRP may be slower than that of water-soluble or ethanol extracts, but its biological activity may be stronger due to the combined dose-dependent effect of active ingredients such as flavonoids and polyphenolic compounds (Liu & Ao, 2021).

Based on the GC–IMS results, two-dimensional topographic plots (Fig. 1 B) and two-dimensional differential topographic plots (Fig. 1 C) were obtained for the volatile compounds. To compare these compounds, drift times were normalized based on the reactive ion peak positions. Most of the signals were at drift times of 1.0–1.5, and the retention times of groups C, L, and M were in the range of 50–200 s, whereas the retention times of group H were delayed to 50–300 s, suggesting that high levels of AMRP accelerated the production and accumulation of volatile compounds. In our previous study, we found that the addition of appropriate levels of flavonoids from AMRP (22 mg/kg diet) significantly improved the flavour of the LT muscle of small-tailed Han sheep (Liu et al., 2019). Therefore, we speculate that the addition of AMRP at different levels may have different effects on the meat quality and flavour of animal products. In our study, AMRP was found to contain volatile compounds with specific aromas, such as alcohols, esters and ketones, which may enrich the flavour of beef by eventually being deposited into beef as calf feed. Additionally, the chances of microbial degradation products of flavonoids and polyphenols entering the animal's hindgut and being deposited in the muscle increase, thus affecting the flavour of the beef, even though these compounds have been reported to have low bioavailability (Mena & Llorach, 2017). Gąsior et al. (2021) reported that the presence of phenolic chemicals in meat may come from beef fed grass and/or grain diets. Raw beef is characterised by a weak odour, but it is rich in non-volatile precursors of volatile compounds responsible for the development of flavour in meat products; it contains amino acids, peptides, and sugars (Williamson et al., 2014). However, reducing sugars and amino acids, as well as peptides, play a key role in the formation of raw meat flavour, which is mainly formed by non-volatile precursor degradation occurring in the beef itself after slaughter, including the degradation of proteins first to peptides and then to amino acids (Meinert et al., 2007) and glycogen degradation to glucose (Meinert et al., 2009). We hypothesise that the antioxidant active components in AMRP have an effect on carbohydrate fermentation, amino acid metabolism, lipid autoxidation, lipid β-oxidation and esterification, and consequently on the concentration of volatile compounds. Ornaghi et al. (2020) reported that the addition of a mixture of natural additives (main components: vanillin, eugenol, and thymol) to the diets of young crossbred (Angus × Nellore) bulls had no significant effect on odour or flavour but was able to improve the overall acceptability of roasted beef. Sheng et al. (2024) reported that shiitake mushroom powder rich in flavonoids and polyphenolic compounds inhibited lipid oxidation in chicken patties and had some effect on the changes in volatile compounds. Guerrero et al. (2018) confirmed our findings in a study where two commercial essential oil mixtures were added at two levels (3.5 g/animal/day and 7.0 g/animal/day) to crossbred (Angus × Nellore) young bull and heifer diets, namely, thyme, eucalyptus and sweet orange). This mixture improved the overall acceptability of beef at a concentration of 3.5 g/animal/day; thus, the type of additive, the level of addition and even the species may have contributed to the differences in the results of the experiment.

#### 3.2.2 Volatile compound analysis of LT

A total of 47 compounds were identified, including 11 ketones (23.40 %), 7 aldehydes (14.89 %), 7 alcohols (14.89 %), 5 amines (10.64 %), 5 esters (10.64 %), 4 acids (8.51 %), 4 heterocycles (8.51 %), and others (8.52 %). Information about these compounds is shown in Supplemental Table 5. Notably, some volatile compounds have protonated monomers (M) and proton-binding dimers (D) that can produce more than two signals at the same time, a property that may be attributed to compounds with high proton affinities or signals that allow the ions to form dimers as they move through the drift tank (Rodríguez-Maecker et al., 2017). Jiang et al. (2024) reported that alcohols, aldehydes and ethers are characteristic flavour compounds of spiced beef and suggested that the production of these compounds is related to the oxidative decomposition of FAs, especially UFAs, and to the involved Maillard reaction. The reason why similar compounds are produced from raw beef may be due to the biochemical reaction of the non-volatile flavour precursors in the sample, as it is subjected to a high temperature of 80 °C in the headspace sampling unit of the gas chromatograph. At high temperatures, reducing sugars and amino compounds can undergo the Maillard reaction (Xie et al., 2022). The Maillard reaction, streak degradation and lipid oxidation play dominant roles in the mechanism of beef flavour formation (Flores, 2018). Ai-Dalali et al. (2022) identified the main sources of volatile flavour substances in marinated raw beef to be alcohols, aldehydes, ethers, furans, and phenols, which are mainly derived from the oxidative decomposition of lipids, the Maillard reaction, thiamine degradation, and metabolite interactions from these reactions.

Several volatile compounds with strong signal intensities were detected in all treatments; the low-RI region (RI < 400) contained undecane (44); the medium-RI region (400 < RI < 800) contained 3-methylbutanal (5), acrylonitrile (46), ethyl acetate (30), 1-penten-3-one (M) (9), 1-penten-3-one (D) (10), acetic acid (34), and tetrahydrofurane (36); and the high-RI region (RI > 800) contained 2-methyl-2-pentenal (M), 2-methyl-2-pentenal (D), 5-methylfurfural, 2-butylfuran, and 2-methylpyrazine, which may be the characteristic flavour compounds of Angus beef. Wan et al. (2023) reported that heptanal, 4-methoxybenzaldehyde, hydroxyacetone, 2,3-butanedione, 3-hydroxy-2-butanone, acetic acid, propionic acid, butyric acid, hexanoic acid, 2,3-butanediol, benzothiazole, γ-butyrolactone and dimethylsulfone were common volatile compounds found in raw beef (*Dzo* cattle), which was similar to the results of our study.

#### 3.2.3 Characteristic volatile fingerprints

**Fig. 2:**
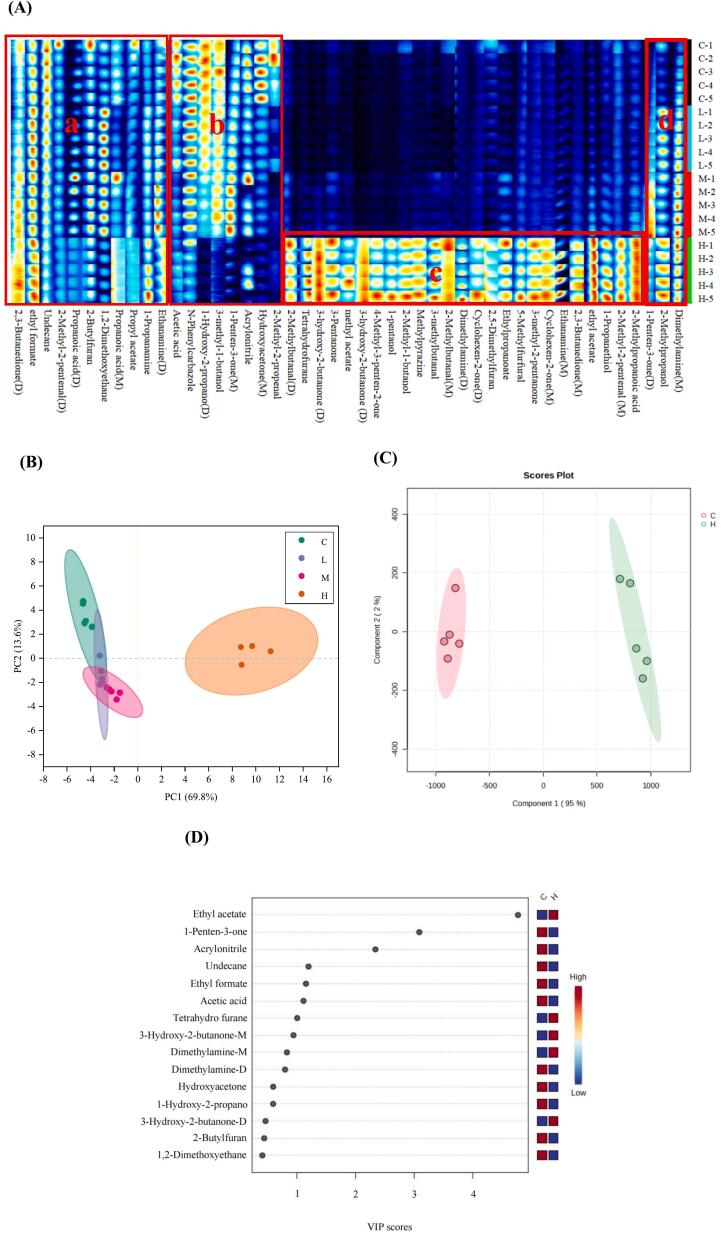

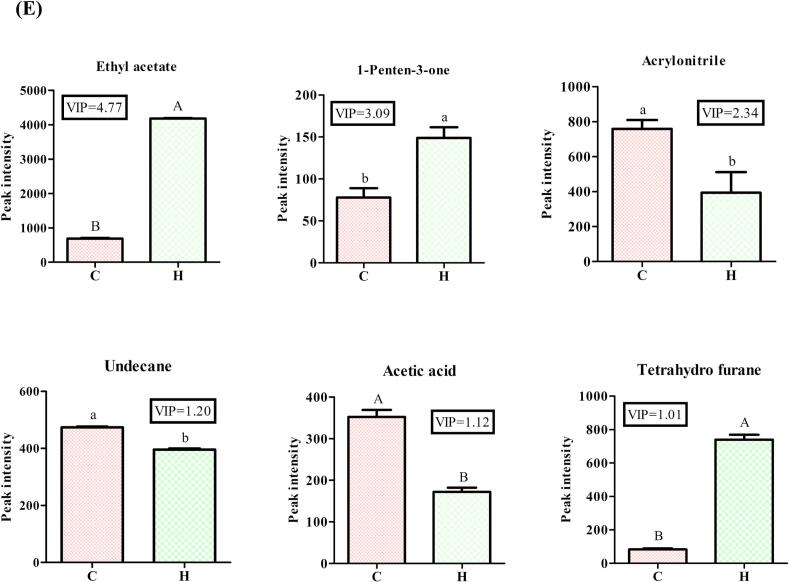
Volatile compound fingerprint (A) and PCA score plots (B) of volatile compounds in the basal diet without additives and after dietary AMRP supplementation of Angus calves in the *longissimus thoracis*. The PLS-DA score (C), VIP score plots (D) of volatile compounds, and peak intensities of ethyl acetate, 1-penten-3-one, acrylonitrile, undecane, acetic acid and tetrahydrofurane (E) in the C group and H group; the screening conditions were VIP ≥ 1 and *P* < 0.05. C, a basal diet without additives; L, M and H indicate 10, 15 or 20 g AMRP per Angus calf per day in the basal diet, respectively. The number of observations for each mean value was five (n = 5). The results are presented as the mean values and the standard error of the mean (SEM). Histogram of the peak intensity () of six characteristic volatile compounds in the LT of the different groups were visualized using the GraphPad Prism 5 package (GraphPad Inc., La Jolla, CA, USA). a-b Mean values with different lowercase letters are significantly different at *P* < 0.05. A-B Mean values with different capital letters are highly significantly different at *P* < 0.01. Note: the bright spot denotes a volatile component in the volatile compound fingerprint, and its hue spans from blue to red, indicating that the concentration of the compound increases. Each row represents all of the signal peaks that were chosen in a sample, and each column depicts the signal peak of the same volatile chemicals in different samples. (For interpretation of the references to colour in this figure legend, the reader is referred to the web version of this article.)

We used a gallery plot to draw characteristic fingerprints corresponding to each treatment, which was used to visually compare the differences in volatile compounds in beef among group C and the L, M, and H groups as well as the specific distribution (Fig. 2 A). Region a shows the 11 volatile compounds present in the four groups of samples, including 2-butylfuran, propanoic acid (D), propanoic acid (M), 2,3-butanedione (D), 2-methyl-2-propenal (D), ethyl formate (M), propyl acetate, 1,2-dimethoxyethane, 1-propanamine, ethanamine-D, and undecane. Region b includes the eight volatile compounds with lower signal intensities in group H beef than in the other groups, including 2-methyl-2-pentenal, hydroxyacetone (M), hydroxyacetone (D), 1-penten-3-one (M), 3-methyl-1-butanol, *N*-phenyl carbazole, acrylonitrile, and acetic acid. Region c contains the 25 volatile compounds with the greatest signal intensity in group H beef: 2-methyl-2-pentenal (M), 5-methylfurfural, 3-methylbutanal, 2-methylbutanal (D), 2-methylbutanal (M), 3-pentanone, 2,3-butanedione (M), cyclohexen-2-one (D), 3-hydroxy-2-butanone (D), 4-methyl-3-penten-2-one, 3-methyl-2-pentanone, cyclohexen-2-one (M), 3-hydroxy-2-butanone, 1-propanethiol, 1-pentanol, 2-methyl-1-butanol, methyl acetate, ethyl propanoate, ethyl acetate, tetrahydrofuran, 2,5-dimethylfuran, methylpyrazine, 2-methylpropanoic acid, ethanamine (D), and dimethylamine (M). The d region indicates a gradual increase in 2-methylpropanol, dimethylamine (M) and 1-penten-3-one (D) signal intensities with increasing levels of AMRP addition, suggesting that the production of these substances is correlated with the level of AMRP addition.

Aldehydes are mainly produced through the degradation and oxidation of lipids and Strecker degradation reactions of amino acids (Dashdorj et al., 2015), and aldehydes usually have a low flavour threshold and can have a notable impact on beef flavour (Watanabe et al., 2015; Zhou et al., 2014). The double bonds in the UFAs in beef are oxidised under certain conditions to produce hydroperoxides, which are readily degraded to other aromatic compounds, especially aldehydes, during heating, and aldehydes produce alcohols under the action of reductases (Zou et al., 2018). The increased abundance of aldehydes, ketones, alcohols and esters in group H suggested that the addition of high levels of AMRP can influence the production of such compounds, which can have a comprehensive effect on the flavour of beef. For example, 3-methylbutanal, 2-methylbutanal (D), and 2-methylbutanal (M) are mainly derived from the metabolism of leucine and isoleucine (Ardo, 2006). Sugars form many volatile compounds when reacting with amino acids, e.g., glucose forms pyrazines with lysine (Ardo, 2006). The volatile compounds in region c gradually increased in variety with increasing levels of AMRP addition, and we hypothesised that this increase might be due to the transfer of flavours unique to AMRP to the meat, as it has been previously reported that *Allium* vegetables contain stilbenoids and organosulfur compounds with stimulating aromas, which have antimicrobial, anti-inflammatory, and antioxidant effects (Baky et al., 2023). Wan et al. (2023) reported that anethole with an aniseed flavour detected in *Dzo* beef may originate from a group of plants grown in Tibet, namely, *Illicium griffithii*, *Pimpinella Tibet*, and *Carum carv*, and that their unique flavour compounds are deposited into the muscle after cattle feeding; they further demonstrated that dietary modifications can enrich beef flavour.

#### 3.2.4 Multivariate statistical analysis of LT

The PCA model identified discrete differences in the volatile compound data points for group C and each *A. mongolicum* Regel treatment group, as shown in Fig. 2 B. The principal components explained 83.4 % of the data variance, and there was a clear trend of separation of volatile compounds in group H from groups C, L and M, suggesting that there was a significant difference between the flavour of beef in group H and the other three groups, whereas the tighter clustering between groups C, L and M suggested that the flavour profile of beef was more similar among these three groups. The results of partial least squares discriminant analysis (PLS-DA) of LT samples from groups C and H are shown in Fig. 2 C. The PLS-DA model explained 97 % of the data variance. In addition, the supervised PLS-DA model was used for both groups, and there was also a clear trend of group separation. The cross validation results of PLS-DA indicate that the model has good classification prediction ability and stability, with R2 = 0.9994 and Q2 = 0.99524.

#### 3.2.5 Screening of characteristic volatile compounds in LT

As shown in Fig. 2D and E, through PLS-DA, a total of 6 characteristic volatile flavour compounds were selected based on the screening conditions of VIP ≥ 1 and *P* < 0.05. Fig. 2E shows that ethyl acetate, 1-penten-3-one, acrylonitrile, undecane, acetic acid and tetrahydrofurane are the most important components influencing the differences in volatile compounds in LT between groups C and H. The contents of ethyl acetate (*P* < 0.01; VIP = 4.77), 1-penten-3-one (*P* < 0.05; VIP = 3.09), and tetrahydrofurane (*P* < 0.01; VIP = 1.01) were significantly greater in the LT in group H than in group C (6.08, 1.91, and 9.11 times, respectively). and those of acrylonitrile, undecane, and acetic acid decreased significantly by 0.52-, 0.83-, and 0.49-fold, respectively.

The content of ethyl acetate was highest in group H, and that of acetic acid was low; moreover, the cellular biochemical reaction of acetic acid activation generates CoA. The accumulation of CoA is essential for the synthesis of ethyl acetate in muscle cells, and alcohol acetyl transferase catalyses the condensation of CoA with ethanol to produce ethyl acetate (Mehta et al., 2020). Ethyl acetate is mainly responsible for fruity, sweet and floral flavours in beef (Dzialo et al., 2017). The threshold of ester compounds is low, so small changes in ester compounds in beef can significantly affect the flavour of beef; therefore, ethyl acetate may be one of the characteristic flavour substances of beef in group H. More importantly, ester synthesis contributes to the regulation of intracellular redox homeostasis (Malcorps & Dufour, 1992), and some esters contribute to the maintenance of plasma membrane fluidity under conditions of stress (Mason & Dufour, 2000), which seems to provide yet another strong piece of evidence for the ability of AMRP to increase the antioxidant potency of muscle cells and reduce stress.

Acetic acid has a strong odour that is pungent, cheesy and vinegary (Hernández-Macedo et al., 2012), and increased levels of acetic acid can lead to undesirable odours in beef. Microbial-mediated fermentation of carbohydrates is the most likely source of organic acids in meat products (Merlo et al., 2021). Mansur et al. (2018) reported that the production of acetic acid was associated with off flavours in beef stored for 21 days under vacuum packaging and was associated with the growth of *L. algidus*, *L. piscium*, *C. divergens* and *Enterobacteriaceae* sp. spoilage organisms, which were positively correlated and could be used as indicators of potential spoilage. Our results showed that AMRP was able to reduce the production of acetic acid in beef. After vacuum packaging of beef, muscle cells are quickly exposed to an anaerobic environment where glycolytic processes dominate and pyruvate produced from glycogenolysis forms CoA and formic acid under the action of lactate dehydrogenase, which in turn generates acetic acid from CoA via the PTA-ACK pathway (Kim et al., 2015). The reduction in acetic acid content in beef in group H may be due to the esterification of acetic acid and alcohols to produce ethyl acetate under nonenzymatic conditions, and the addition of AMRP can reduce the accumulation of acetic acid in beef and promote its conversion to ethyl acetate or its participation in other biochemical reactions, which can improve the flavour of beef.

The UFAs generated by lipid hydrolysis can produce many aldehydes, alcohols and ketones through oxidative degradation by heat, of which ketones are also important factors in the formation of beef flavour. Ai-Dalali et al. (2022) predicted potential precursors and metabolic pathways of major flavour components using the MetaCyc platform and confirmed that the major volatile compounds in raw beef are mainly produced through the metabolism of amino acids and FAs. Our previous study revealed that the addition of 20 g/d/calf AMRP to the diet significantly increased the LT C18:1n9c ratio, MUFA content, and UFA:SFA ratio in Angus calves and that AMRP supplementation may be an effective strategy for improving the health value of beef lipids (Liu et al., 2024); thus, increasing the MUFA content promotes the generation of characteristic beef flavours. Wu et al. (2020) reported that MUFAs, such as oleic acid C18:1n9c, can be enzymatically oxidised or autoxidised to produce hydroperoxides such as 8-hydroperoxide, 9-hydroperoxide, 10-hydroperoxide, 11-hydroperoxide and 13-hydroperoxide, which are degraded to aldehydes by homolytic cleavage, and aldehydes, alcohols and ketones are interconvertible.

Furans come from a variety of sources and are usually produced during carbohydrate catabolism due to lipid oxidation and amino acid catabolism (Merlo et al., 2021); notably, furan formation is related to the pH of the environment, with furan synthesis reactions being enhanced under alkaline or neutral conditions (Batool et al., 2020). Many of these compounds are known to have a caramel-like aroma (Shahidi et al., 2004). Alkanes form the basis of the hydrocarbon chain of SFAs, and it was hypothesised that the significantly lower content of undecane in the samples of group H might be related to the low content of SFAs. Overall, our results revealed that the characteristic volatile compounds screened may originate from different lipid compound precursors in each group, and thus, we will resolve the mechanisms responsible for the flavour differences between the two groups of samples from a lipidomic perspective.

### 3.3 Lipid compound analysis of the LT from calves in different treatment groups

#### 3.3.1 Lipid composition of the LT

**Fig. 3:**
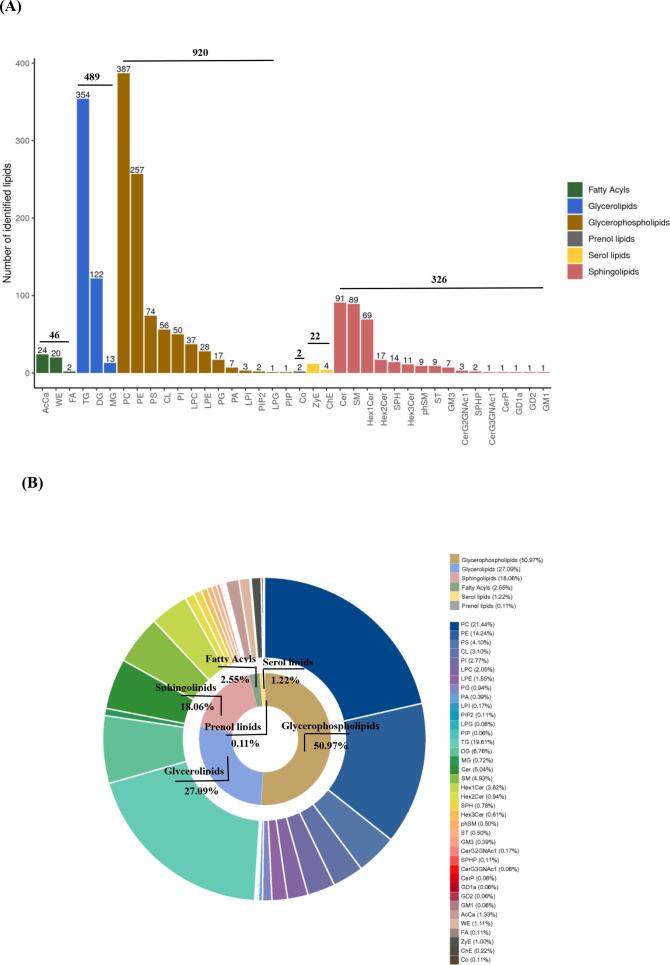

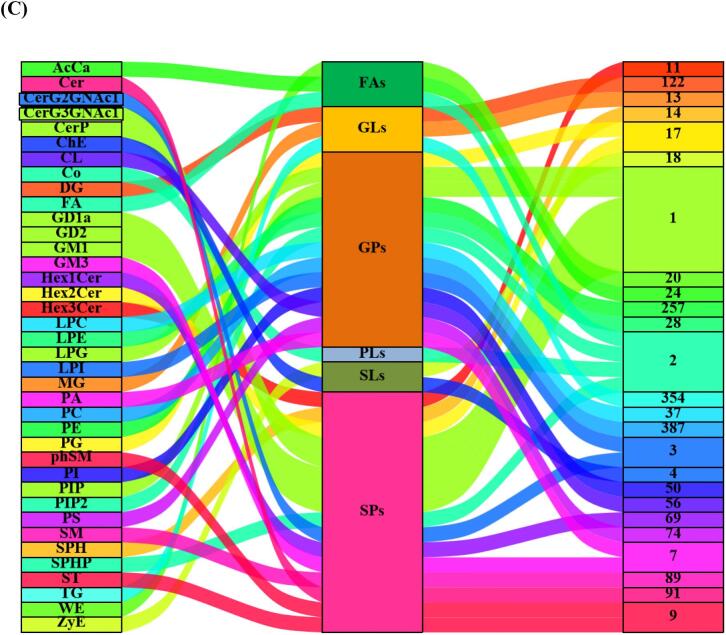
Numbers of lipid species identified in 6 classes and 38 lipid subclasses (A). Percentages of each lipid subclass and class (B). Sankey diagram of 1805 lipid compounds (C).

LC–MS was used to identify 1805 lipid compounds from group C and H beef samples in positive and negative ionisation modes. As shown in Fig. 3A and B, the detected compounds were classified into 38 lipid subclasses, including 387 (21.44 %) PCs, 354 (19.61 %) TGs, 257 (14.24 %) PEs, 122 (6.76 %) DGs, 91 (5.04 %) ceramides (Cers), 89 (4.93 %) sphingomyelins (SMs), 74 (4.10 %) phosphatidylserines (PSs), 69 (3.82 %) hexosyl ceramides (Hex1Ces), 56 3.10 % cardiolipins (CL), 50 (2.77 %) PIs, 37 (2.05 %) lysophosphatidylcholines (LPCs), 28 (1.55 %) lysophosphatidylethanolamines (LPEs), 24 (1.33 %) acylcarnitines (AcCas), 20 (1.11 %) wax esters (WEs), 18 (1.00 %) zymosterols (ZyEs), 17 (0.94 %) hexosyl ceramides (Hex2Cers), 17 (0.94 %) phosphatidylglycerols (PGs), 14 (0.78 %) sphingosine bases (SPHs), 13 (0.72 %) monoglycerides (MGs), 11 (0.61 %) hexosyl ceramides (Hex3Cers), 9 (0.50 %) phytosphingosines (phSMs), 9 (0.50 %) sulfatides (STs), 7 (0.39 %) gangliosides (GM3), 7 (0.39 %) phosphatidic acids (PAs), 4 (0.22 %) cholesterol esters (ChEs), 3 (0.17 %) *N*-acetylhexosyl ceramides (CerG2MNAc1s), 3 (0.17 %) lysophosphatidylinositols (LPIs), 2 (0.11 %) coenzyme Qs (Cos), 2 (0.11 %) FAs, 2 (0.11 %) sphingosine phosphates (SPHPs), 2 (0.11 %) phosphatidylinositol(4,5)bisphosphates (PIP2s), 1 (0.08 %) dihexosyl *N*-acetylhexosyl ceramide (CerG3GNAc1), and 6 (0.30 %) others. Notably, PCs, TGs and PEs determine the abundance of lipid compounds in beef. As shown in Fig. 3B and C, all the lipids were further classified into six major groups, i.e., 920 (50.97 %) glycerophospholipids (GPs), 489 (27.09 %) glycerolipids (GLs), 326 (18.06 %) sphingolipids (SPs), 46 (2.55 %) fatty acyls, 22 (1.22 %) serol lipids (SLs) and 2 (0.11 %) prenol lipids (PLs). Notably, GPs and GLs had the highest contents in beef, which is in agreement with the results of Maritha et al. (2023). TGs are the most important form of lipids in plants and animals, and their breakdown to form free FAs is essential for flavour formation. Xiong et al. (2022) reported that FAs in yak *longissimus dorsi* were mainly in the form of TGs and PCs, which was consistent with our results. TGs can be further hydrolysed to produce free FAs, which are the key to the formation of beef flavour, and the oxidation of PCs produces a large amount of volatile flavouring substances, which give beef a unique flavour. This is consistent with our previous findings that the addition of 20 g/d/Calf of AMRP to the feed significantly increased C18:1n-9 and C18:2n-6 levels in LT and improved the fatty acid composition of Angus beef (Liu et al., 2024).

#### 3.3.2 Multivariate statistical analysis of LT

**Fig. 4:**
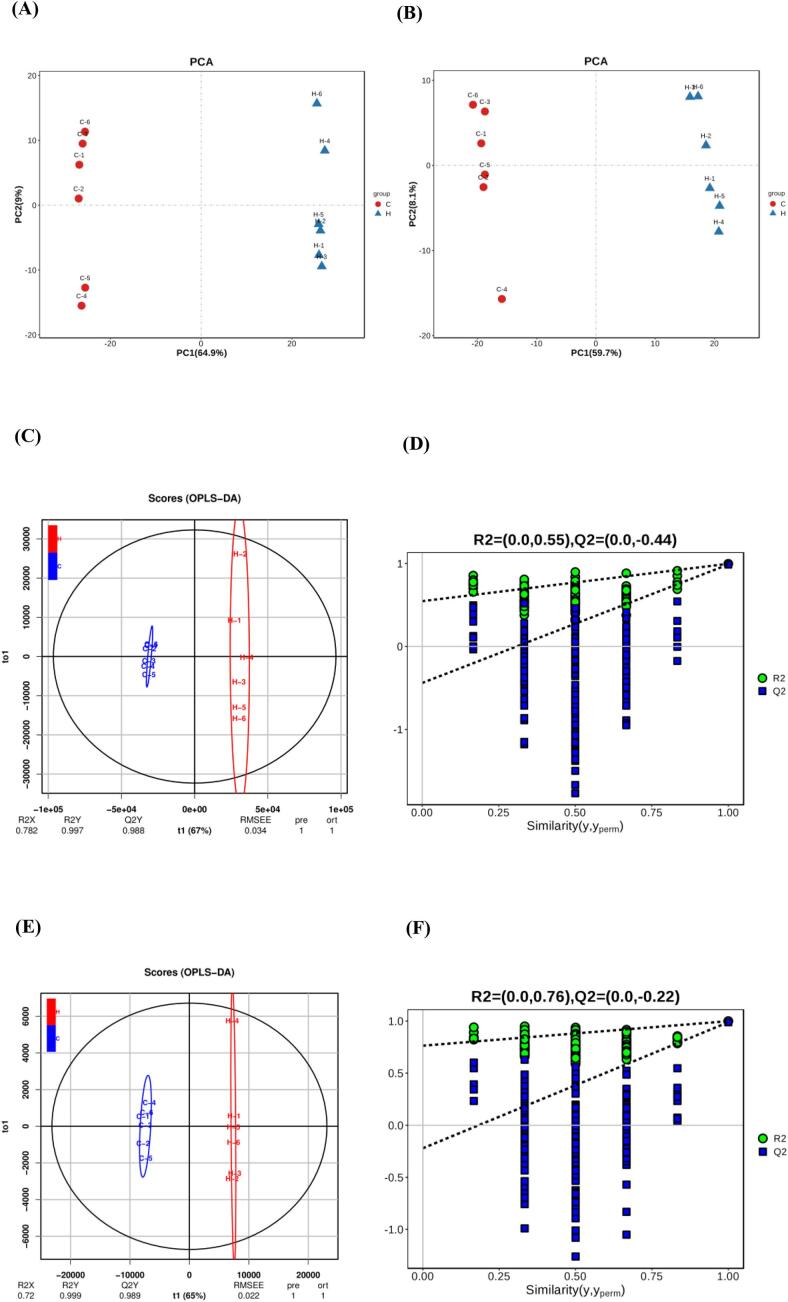

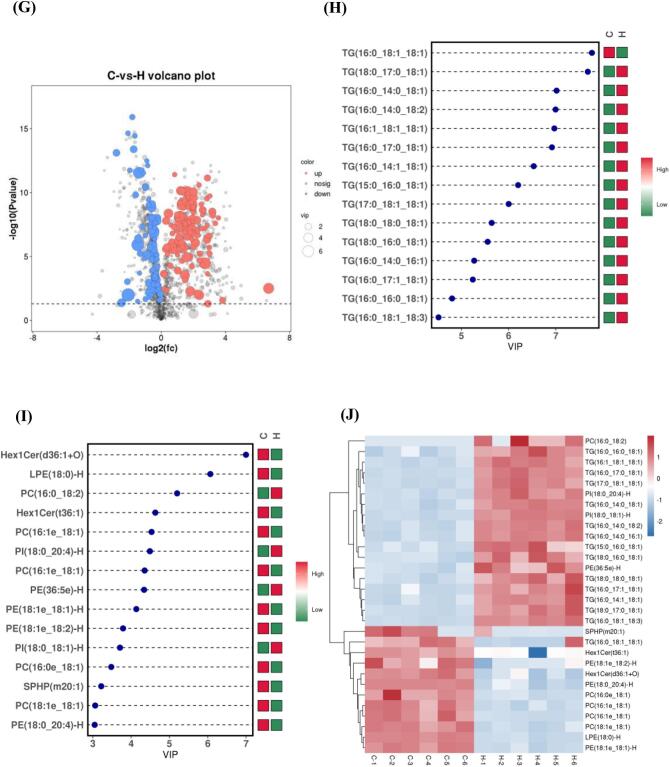
The PCA score plots of test and quality control (QC) samples in positive (A) and negative (B) ionisation modes. OPLS-DA score plots (C) and corresponding OPLS-DA validation (D) of the test samples in positive ionisation mode. OPLS-DA score plots (E) and corresponding OPLS-DA validation (F) of the test samples in negative ionisation mode. Volcano map of all differentially abundant metabolites of the differentially abundant lipid metabolites of *longissimus thoracis* samples in the C and H groups (G). VIP score plots of OPLS-DA in the positive (H) and negative (I) ionisation modes; the screening conditions were VIP ≥ 1 and *P* < 0.05. The heatmap (J) of the key lipid metabolites of *longissimus thoracis* samples in the C and H groups. C, basal diet without additives; H, 20 g AMRP per Angus calf per day supplemented in the basal diet. The number of observations for each mean value was six (*n* = 6).

To gain a deeper understanding of the overall distribution of lipids in LT samples from calves in groups H and C and to screen differentially abundant lipid metabolites, we subjected the lipid identification data to a multilevel statistical analysis model, including one-way statistical analyses (Student's *t*-test and collapsed change) and multifactorial statistical analyses (unsupervised PCA and supervised OPLS-DA). PCA identified discrete differences in lipid metabolite data points in group C and H beef. As shown in Fig. 4A and B, PCA explained 71.2 % and 63.8 % of the data variance in the positive and negative ionisation modes, respectively, and there was a clear trend of separation between groups C and H, suggesting that the lipid metabolite profiles differed between the two groups. In contrast to the PCA model, the OPLS-DA model introduced grouping variables and filtered out noise unrelated to the categorical information, and subsequent model tests and differentially abundant lipid metabolite screening were subsequently performed using the OPLS-DA results. This method can effectively reduce the complexity of the model and enhance the explanatory ability of the model without reducing the predictive ability of the model to maximise the differences between group C and group H and improve the parsing ability and validity of the model. As shown in Fig. 2C and E, t1 maximisation reflects the between-group differences, which further suggests that the lipid composition of groups C and H changed differently under AMRP intervention in the positive and negative ionisation modes, consistent with the results of PCA. In addition, we examined the prediction accuracy and goodness-of-fit of the OPLS-DA model through 7 rounds of cyclic interactive validation and 200 response sequencing tests. As shown in Fig. 2D and F, the R2 and Q2 values of 0.55 and − 0.44, respectively, in positive ionisation mode and 0.76 and − 0.22, respectively, in negative ionisation mode confirm that the analytical model is reliable and does not exhibit overfitting. As shown in the volcano plot in Fig. 2G, a total of 199 differentially abundant lipid metabolites were identified in groups C and H, of which 112 were upregulated (*P* < 0.05; VIP > 1) and 87 were downregulated (*P* < 0.05; VIP > 1) (Supplemental Table 6). The main components were 33.67 % TGs, 20.60 % PCs, 12.56 % PEs, 7.54 % DGs, 4.02 % PIs, 3.52 % LPCs, 3.52 % LPEs, 2.51 % ZyEs, 2.51 % Cers, 2.01 % PSs, 2.01 % Hex1Cers, 1.51 % SMs, 1.01 % SPHs, 0.50 % MGs, 0.50 % AcCas, 0.50 % STs, 0.50 % CLs, 0.5 % phSMs, 0.50 % MGs, 0.50 % AcCas, 0.50 % STs, 0.50 % CLs, 0.5 % phSMs, and 0.50 % SPHPs.

#### 3.3.3 Identification of key lipid metabolites

In this study, the screening criteria for key lipid metabolites between groups C and H were based on the variable weighting (VIP > 1) and *t*-test (*P* < 0.05) of the OPLS-DA model. The top 15 key lipid metabolites screened in the positive and negative ionisation modes are shown in Fig. 2H and I, respectively. As shown in Supplemental Table 7, the 30 key lipid metabolites included 50.00 % TGs, 16.67 % PCs, 13.33 % PEs, 6.67 % Hex1Cers, 6.67 % PIs, 3.33 % LPEs, and 3.33 % SPHPs.

Furthermore, based on the hierarchical cluster analysis method, we intuitively showed and analysed the variance between the C and H samples as well as the expression abundance of key lipids in the different samples. Fig. 4 J shows the distribution of the contents of 30 key lipid metabolites in the samples of the two groups, with higher levels of most of the differentially abundant metabolites in the H group. The distribution of the levels of each differentially abundant metabolite in the two groups was consistent with the results of VIP score plot analysis. Notably, we identified 30 key lipid metabolites with a total carbon number between 21 and 57, a double bond number distribution between 0 and 4, and a predominance of long-chain MUFAs. All 14 TGs of group H were highly significantly upregulated, except for TG (16:0_18:1_18:1), which was highly significantly downregulated (*P* < 0.01). The hydrolysis of lipids is mainly the hydrolysis of TGs, and due to the large upregulation of TGs in group H, we hypothesised that significant hydrolysis was experienced in the muscle samples and that muscle TGs can be oxidatively catabolised to provide energy when exogenous fats are deficient or insufficiently ingested (Jia et al., 2021). TGs in organisms are hydrolysed to free fatty acids by lipases along with glycerol, which undergoes a series of enzyme-catalysed reactions to enter the glucose metabolic pathway or other metabolic pathways (Segura et al., 2015). For PCs, except for PC (16:0_18:2), which was significantly upregulated (*P* < 0.05), PC (16:1e_18:1), PC (16:1e_18:1), PC (16:0e_18:1), and PC (18:1e_18:1) were all significantly downregulated (*P* < 0.05). In the PE subclass, except for PE (36:5e)-H, which was significantly upregulated (*P* < 0.05), PE (18:1e_18:1)-H, PE (18:1e_18:2)-H and PE (18:0_20:4)-H, were all highly significantly downregulated (*P* < 0.01). Phospholipid molecules are important components of cell membranes and are classified according to their functional head groups into PA, PC, PE, PI, PG, PS and lysophosphatidyl forms such as LPC and LPE (Colin & Jaillais, 2020). PCs are important components of biofilms and have an emulsifying effect on lipid removal. When PC and PE accumulate in large amounts, lipid oxidation is easily triggered, and the downregulation of PEs may be further involved in the Maillard reaction of reducing sugars (e.g., d-glucose) rather than hydrolysis, as quantum chemical calculations have shown that the Maillard reaction energy for PEs (96.81 kJ/mol) is much lower than that for PE hydrolysis (188.84 kJ/mol) (Zhang et al., 2023). Group H beef may contain large amounts of reducing sugars such as glucose, fructose, arabinose, and xylose, which provide a carbon source for the Maillard reaction. PI (18:0_20:4)-H was highly significantly upregulated, and PI (18:0_18:1)- was highly significantly upregulated. Zhou et al. (2021) showed that SFAs, PIs, and PSs may contribute to IMF deposition, which is a key factor for the flavour of meat regarding IMF, as shown by the results of lipidomic analysis. In this study, PI(18:0_20:4)-H and PI(18:0_18:1)- were significantly upregulated, and Zhou et al. (2021) suggested that SFAs, PIs, and PSs might contribute to the deposition of IMF according to the results of lipidomics analysis, which is a key factor that affects the flavour of meat regarding IMF. The endoplasmic reticulum lipid composition and membrane tension are key parameters for the formation of lipid droplets, which are intracellular emulsion droplets that regulate cellular energy metabolism, and almost all cells and organisms are able to convert excess nutrients into lipid droplets (Ben M'barek et al., 2017). Phospholipids are essential for endoplasmic reticulum completeness and normal function. Inositol is a major component of phospholipid synthesis and is the precursor for the synthesis of PI, the major lipid in endoplasmic reticulum membranes. The addition of inositol leads to a 5-fold increase in the levels of PI and overall cytosolic phospholipids, and the production of phospholipids salvages aberrant endoplasmic reticulum surfaces and accelerates the formation of lipid droplets (Chorlay et al., 2019). LPE (18:0)-H was highly significantly downregulated in group H. PEs and LPEs could be used as biomarkers to differentiate between patients with Alzheimer's disease and a healthy population (Llano & Devanarayan, 2021).

### 3.4 Correlation analysis of key lipid compounds and characteristic volatile compounds

**Fig. 5:**
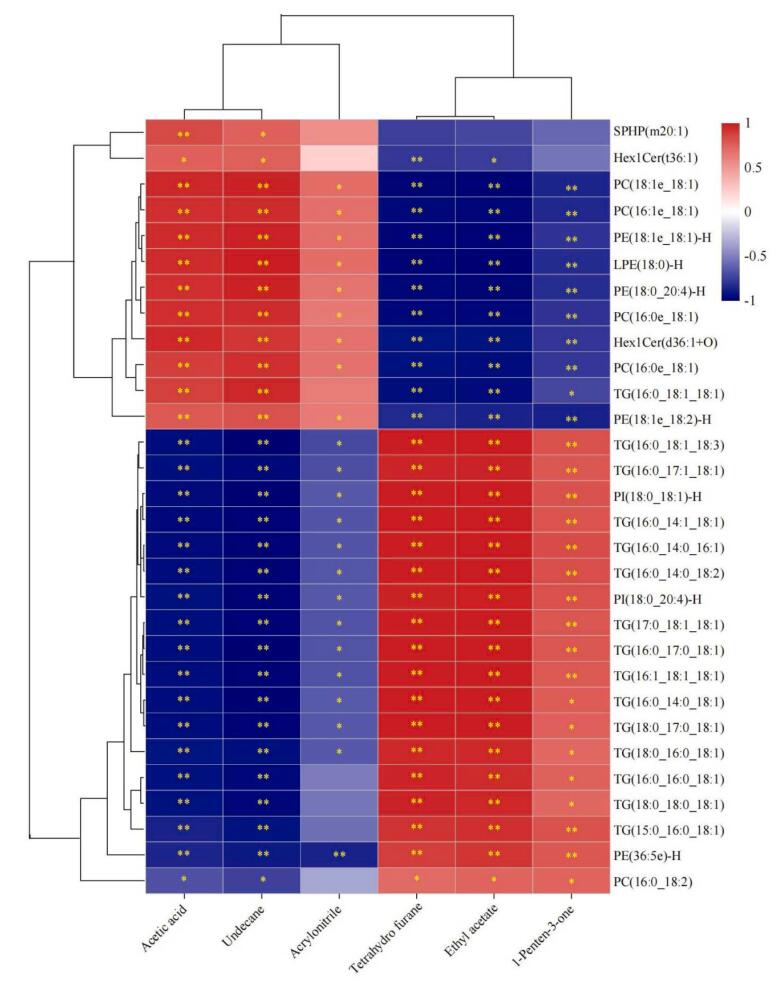
Spearman's correlation heatmap showing the correlation between key lipid compounds and characteristic volatile compounds. The colour represent correlation coefficients, with red indicating a positive correlation and blue indicating a negative correlation. ^⁎^ and ^⁎⁎^ represent significance at *P* < 0.05 and *P* < 0.01, respectively. (For interpretation of the references to colour in this figure legend, the reader is referred to the web version of this article.)

**Fig. 6:**
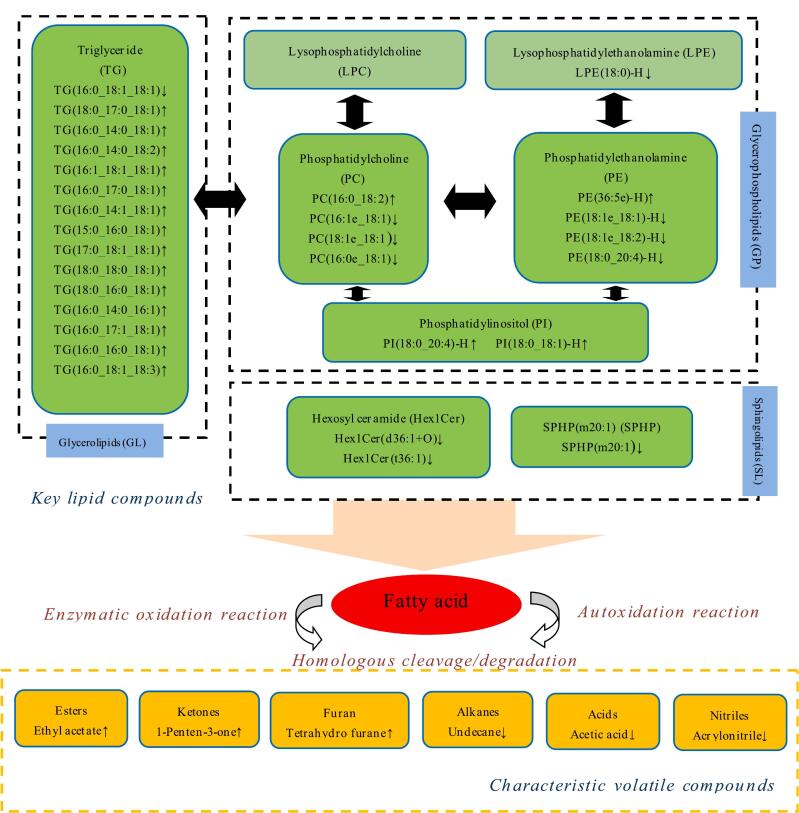
Potential relationship between key lipid compounds and characteristic volatile compounds.

To further understand the effect of lipid degradation and oxidation on the formation of volatile compounds, 30 key lipid metabolites and 6 characteristic volatile compounds were selected for correlation analysis, and the results are shown in Fig. 5. Ethyl acetate, 1-penten-3-one and tetrahydrofurane showed significant positive correlations with most of the TGs and PIs except TG (16:0_18:1_18:1) and significant negative correlations with most of the PCs, PEs and LPEs, whereas the opposite was the case for the other three characterised volatile compounds acetic acid, undecane and acrylonitrile. Tetrahydrofurane showed a highly significant positive correlation with TG (16:0_14:0_18:1) (ρ = 0.994; *P* = 0.000) and a highly significant negative correlation with LPE (18:0)-H (ρ = −0.989; *P* = 0.000). Ethyl acetate showed a highly significant positive correlation with TG (16:0_18:1_18:3) (ρ = 0.997; *P* = 0.000) and a highly significant negative correlation with LPE (18:0)-H (ρ = −0.996; *P* = 0.000). 1-Penten-3-one showed a highly significant positive correlation with TG (16:0_14:0_16:1) (ρ = 0.863; *P* = 0.003) and a highly significant negative correlation with PE (18:1e_18:2)-H (ρ = −0.869; *P* = 0.001). Acetic acid showed a highly significant positive correlation with PC(18:1e_18:1) (ρ = 0.962；*P* = 0.000) and a highly significant negative correlation with TG(16:0_14:1_18:1) (ρ = −0.964；*P* = 0.000). Undecane showed a highly significant positive correlation with LPE(18:0)-H (ρ = 0.985；*P* = 0.000) and a highly significant negative correlation with PI(18:0_18:1)-H (ρ = −0.996；*P* = 0.000). Acrylonitrile showed a significant positive correlation with PC(18:1e_18:1) (ρ = 0.699；*P* = 0.024) and a highly significant negative correlation with PE(36:5e)-H (ρ = −0.865；*P* = 0.001) (Supplemental Table 8). There is direct evidence that the production of volatile compounds such as aldehydes, alcohols, ketones and furans is directly related to lipid autoxidation, specifically these substances are primary oxidation products from lipid autoxidation, conjugated olefinic compounds, and secondary oxidation products from the further breakdown of lipid hydroperoxides (Xu et al., 2023). Numerous studies have reported that UFAs can produce a large amount of volatile flavour compounds, including aldehydes, alcohols, ketones, esters and heterocyclic compounds (Van et al., 2013; Zhou et al., 2014). In the major connectivity panorama of key lipid metabolites and characteristic volatile compounds (Fig. 6), it is clear that most of the TGs are up-regulated, and furthermore, we hypothesised that the production of ketones, esters and furans in the characteristic flavour substances of the H group was mainly related to UFAs in TGs rather than UFAs in PEs and PCs, which have greater thermo-oxidative stability at the *s*n-2 site in PCs and PEs than in TGs (Zhou et al., 2014). This finding is similar to the findings of Xiong et al. (2022), who suggested that changes in volatile flavour substances in yak muscle were mainly caused by UFAs (i.e. TG (18:1_18:1_18:2), PC (18:2_18:0), TG (16:1_18:1_18:1), and PI (18:0_20:4). Zheng et al. (2024) reported that most of the ketones in Nuodeng ham are produced by reactions associated with monounsaturated fatty acids. Ketones are mainly derived from lipid oxidation, amino acid degradation and melting reactions and have an aromatic flavour (Zhang et al., 2019). Sulfur-free furans are derived from oxidative degradation products of oleic acid and are predominantly sweet, nutty and caramelised (Madruga et al., 2010). In this experiment, ethyl acetate, 1-penten-3-one and tetrahydrofurane were important markers of the flavour profile in group H. These compounds were associated with UFAs in TGs, and the addition of AMRP significantly improved beef flavour. In addition, in subsequent studies we should consider that the presence of hydrophobic, van der Waals, hydrogen and ionic bonding interactions between volatile compounds and matrices can affect the natural release of flavouring substances from matrices to the environment (Xu et al., 2024). The contribution of individual volatile compounds to the formation of characteristic beef flavours varies and it is therefore extremely important to separate these characteristic volatile compounds from other flavourless food components. Further studies are needed to provide additional evidence to confirm the observed associations and to elucidate the key lipid molecules associated with flavour and their detailed regulatory pathways. We also should further explore the toxicological response and metabolism of the active ingredients in AMRP in the animal organism, especially the modification of the active ingredients and their interaction with the host mediated by gut microbes.

## 4 Conclusion

This study is the first to examine the contribution of altered lipid composition of the LT of Angus calves to the formation of lipogenic trait volatile compounds following dietary supplementation with AMRP from a multi-omics perspective of lipidomics and volatile compounds. A total of 47 volatile compounds were detected, of which ethyl acetate, 1-penten-3-one, acrylonitrile, undecane, acetic acid and tetrahydrofurane were identified as the characteristic volatile compounds of beef in the AMRP treatment group ethyl acetate, and 1-penten-3-one and tetrahydrofurane were significantly upregulated and improved beef flavour. A total of 199 differentially abundant lipid metabolites were identified by lipidomics, of which 112 were upregulated and 87 were downregulated, and the key differential lipid compounds upregulated in the AMRP treatment group were mainly concentrated in the TG and PI subclasses. The results of the correlation analysis revealed that AMRP may affect the TG composition and metabolite production in beef lipids to improve beef flavour. These findings provide a scientific and theoretical basis for the development and utilisation of AMRP as a feed additive for the overall improvement of beef flavour. Future studies will focus on the molecular level in conjunction with cell culture to investigate the regulatory effects of AMRP on IMF metabolism and flavour in Angus calves.

## Funding

This work was supported by Gansu Provincial Department of Education, Industrial Support Program Project (grant no. 2024CYZC-36), the National Natural Science Foundation of China (grant no. 32260846; 32402789), the Discipline Team Project of Gansu Agricultural University (GAU-XKTD-2022-24), and Aoxin Farming (Tianjin) Co., Ltd., R&D Special (grant no. GSAU-JSYF-2021-025).

## CRediT authorship contribution statement

**Wangjing Liu:** Methodology, Investigation, Funding acquisition, Formal analysis, Data curation, Conceptualization. **Huixia Gao:** Formal analysis, Data curation. **Jianjian He:** Formal analysis, Data curation. **Aihuan Yu:** Formal analysis. **Chenxu Sun:** Methodology. **Yaodi Xie:** Resources. **Haibo Yao:** Resources. **He Wang:** Resources. **Yueyan Duan:** Resources, Project administration. **Jinsheng Hu:** Methodology. **Zhaomin Lei:** Resources. **Defu Tang:** Conceptualization.

## Declaration of competing interest

No conflicts of interest exist in the submission of this manuscript, and the manuscript is approved by all authors for publication. The work described is original research that has not been published previously and is not under consideration for publication elsewhere, in whole or in part. All the listed authors have approved the enclosed manuscript.

## Data Availability

Data will be made available on request.

## Appendix A Supplementary data

Supplementary material Image 1
