# Supplementary material for: Evaluation of the effect of dietary supplementation with Allium mongolicum regel bulb powder on the volatile compound and lipid profiles of the longissimus thoracis in Angus calves based on GC–IMS and lipidomic analysis
Source: Food Chem X. 2024 Sep 10;24:101820. doi: 10.1016/j.fochx.2024.101820 (PMC11459021; doi:10.1016/j.fochx.2024.101820)
Supplement: Supplementary file 1 — Supplementary material [file mmc1.docx]

**Supplemental Figure**

**(A)**


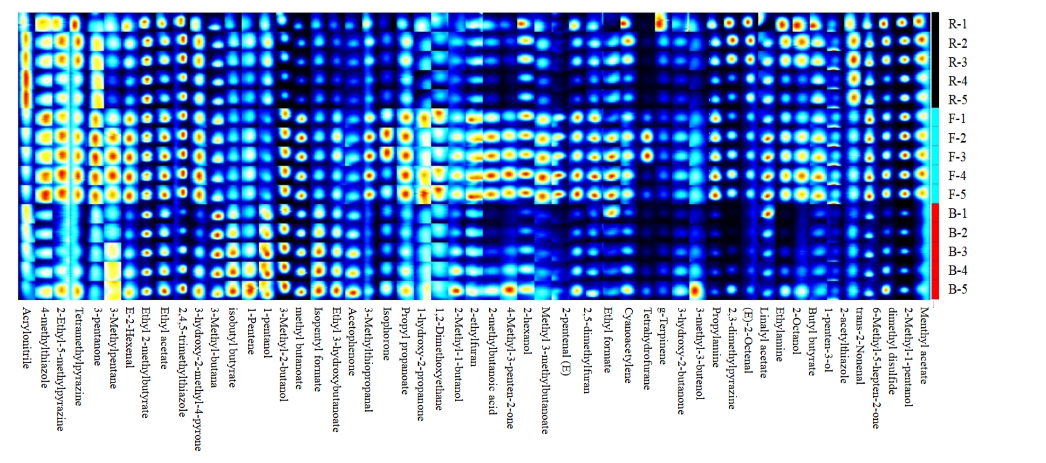


**(C)**

**(B)**


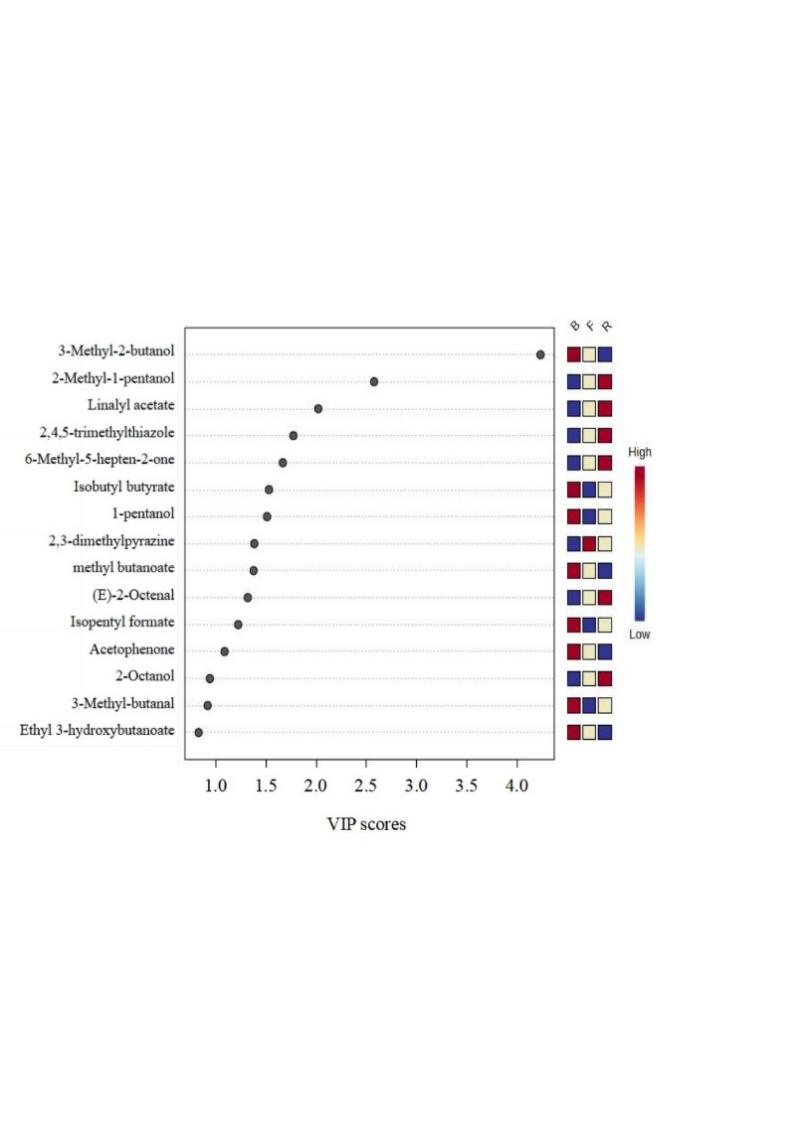


Supplemental Figure 1. Volatile compound fingerprints (A), PCA score plots (B) and VIP score plots (C) of volatile compounds in the roots, bulbs and flowers of *Allium mongolicum* Regel. R, roots of *A. mongolicum* Regel; B, bulbs of *A. mongolicum* Regel; F, flowers of *A. mongolicum* Regel. The number of observations for each mean value was five (n = 5).

**Supplemental Tables**

**Supplemental Table 1**

The dietary ingredients, nutrient levels, and fatty acid composition of the basal diets (DM basis, %)

| Items | Fattening stages | | |
| --- | --- | --- | --- |
|  | Phase I (Days 1 to 60) | | Phase II (Days 61 to 120) |
| Ingredients |  |  | |
| Corn silage | 16.66 | 12.59 | |
| Soybean meal | 6.63 | 15.02 | |
| Guinea grass | 19.54 | 19.69 | |
| Rice straw | 38.31 | 28.95 | |
| Corn | 6.29 | 7.92 | |
| Wheat bran | 5.51 | 7.14 | |
| Corn germ meal | 5.56 | 7.19 | |
| Premix^1)^ | 1.50 | 1.50 | |
| Total | 100.00 | 100.00 | |
| Nutrient levels |  |  | |
| NE_m_/(KJ/kg) ^2)^ | 5.63 | 6.12 | |
| NEg/(KJ/kg) ^3)^ | 2.63 | 3.20 | |
| Ash | 12.60 | 10.04 | |
| Crude protein | 11.84 | 11.02 | |
| Ether extract | 2.20 | 2.65 | |
| Neutral detergent fibre | 48.60 | 50.27 | |
| Acid detergent fibre | 29.60 | 30.09 | |
| Ca | 0.81 | 0.50 | |
| P | 0.21 | 0.36 | |
| Fatty acids (mg/g) |  |  | |
| C13:0 | 0.50 | 0.80 | |
| C14:0 | 0.70 | 1.00 | |
| C16:0 | 16.00 | 17.00 | |
| C16:1 | 0.10 | 0.15 | |
| C18:0 | 2.30 | 2.56 | |
| C18:1*n*9c | 9.12 | 9.77 | |
| C18:2*n*6c | 7.15 | 7.54 | |
| C18:3*n*3 | 1.78 | 2.00 | |
| C20:5*n*3 | 0.80 | 0.84 | |
| C22:6*n*3 | 0.10 | 0.21 | |
| C22:0 | 0.52 | 0.54 | |
| C24:0 | 0.34 | 0.32 | |

Note: ^1)^ Contained (per kg): 950 IU of vitamin A, 180 IU of vitamin D, 25 IU of vitamin E, 10 mg of Cu, 80 mg of Fe, 20 mg of Mn, 40 mg of Zn, 1.0 mg of I, and 0.6 mg of Se. ^2)^ The net energy for maintenance, ^3)^ the net energy for gain; NEm and NEg were calculated according to NASEM (2016)

**Supplemental Table 2**

Nutrients contents, and fatty acid composition of *Allium mongolicum* Regel bulb leaves (% dry matter basis)

| Items | Numerical value |
| --- | --- |
| Nutrients contents | |
| Crude protein | 30.75 |
| Crude fat | 5.27 |
| Neutral detergent fiber | 18.57 |
| Acidic detergent fiber | 15.82 |
| Ca | 0.97 |
| P | 0.58 |
| Fatty acids (mg/g) |  |
| C12:0 | 0.06 |
| C15:0 | 0.02 |
| C16:0 | 0.17 |
| C16:1 | 0.05 |
| C17:0 | 0.02 |
| C18:0 | 0.09 |
| C18:1*n*9c | 0.02 |
| C18:1*n*7c | 0.02 |
| C18:2*n*6c | 0.09 |
| C18:3*n*3c | 1.80 |
| C18:3*n*6c | 1.90 |
| C20:0 | 0.26 |
| C20:1 | 0.02 |
| C20:2 | 0.04 |
| C21:0 | 0.10 |
| C22:0 | 0.30 |
| C23:0 | 0.05 |
| C24:0 | 0.13 |

**Supplemental Table 3**

Identification of 27 volatile compounds in bulb of *Allium mongolicum* Regel using gas chromatography–ion mobility mass spectrometry (GC –IMS).

| No. | Compound | CAS | Molecule formula | MW^1)^ | RI^2)^ | RT/s^3)^ | DT/s^4)^ | Odour description |
| --- | --- | --- | --- | --- | --- | --- | --- | --- |
|  |  |  |  |  |  |  |  |  |
| Aldehydes | | | | | | | | |
| 1 | E-2-Hexenal | C6728263 | C_6_H_10_O | 98.1 | 823.5 | 226.192 | 1.16066 | Green |
| 2 | 3-Methyl-butanal | C590863 | C_5_H_10_O | 86.1 | 695 | 162.529 | 1.18743 | Fruity |
| Ketones | | | | | | | | |
| 3 | 3-Pentanone | C96220 | C_5_H_10_O | 86.1 | 683.3 | 158.237 | 1.10098 | Cheesy |
| 4 | Acetophenone | C98862 | C_8_H_8_O | 120.2 | 1101.7 | 503.003 | 1.18706 | Floral |
| 5 | 4-Methyl-3-penten-2-one | C141797 | C_6_H_10_O | 98.1 | 807.6 | 215.741 | 1.46629 | Floral |
| Alcohols | | | | | | | | |
| 6 | 3-Hydroxy-2-methyl-4-pyrone | C118718 | C_6_H_6_O_3_ | 126.1 | 1077.5 | 468.172 | 1.14357 | Caramel |
| 7 | 1-Pentanol | C71410 | C_5_H_12_O | 88.1 | 751.1 | 186.35 | 1.24806 | Spicy |
| 8 | 3-Methyl-2-butanol | C598754 | C_5_H_12_O | 88.1 | 627.2 | 141.499 | 1.43352 | Fermented |
| 9 | 1-Hydroxy-2-propanone | C116096 | C_3_H_6_O_2_ | 74.1 | 647.3 | 147.508 | 1.2623 | Buttery |
| 10 | 2-Methyl-1-butanol | C137326 | C_5_H_12_O | 88.1 | 725.8 | 175.62 | 1.2329 | Floral/Fruity |
| 11 | 2-Hexanol | C626937 | C_6_H_14_O | 102.2 | 795.7 | 207.908 | 1.30599 | Sulfurous |
| Esters | | | | | | | | |
| 12 | Methyl butanoate | C623427 | C_5_H_10_O_2_ | 102.1 | 695.5 | 162.744 | 1.44511 | Fruity |
| 13 | Ethyl 2-methylbutyrate | C7452791 | C_7_H_14_O_2_ | 130.2 | 841.6 | 238.078 | 1.263 | Fruity |
| 14 | Propyl propanoate | C106365 | C_6_H_12_O_2_ | 116.2 | 822.7 | 225.663 | 1.22754 | Fruity |
| 15 | Isobutyl butyrate | C539902 | C_8_H_16_O_2_ | 144.2 | 950.7 | 316.336 | 1.34262 | Fruity |
| 16 | Ethyl 3-hydroxybutanoate | C5405414 | C_6_H_12_O_3_ | 132.2 | 919.1 | 292.037 | 1.19245 | Fruity |
| 17 | Isopentyl formate | C110452 | C_6_H_12_O_2_ | 116.2 | 801.2 | 211.563 | 1.26676 | Fruity |
| 18 | Ethyl acetate | C141786 | C_4_H_8_O_2_ | 88.1 | 626.5 | 141.284 | 1.32741 | Fruity |
| Heterocycles | | | | | | | | |
| 19 | Tetramethylpyrazine 2,3,5,6 | C1124114 | C_8_H_12_N_2_ | 136.2 | 1075.5 | 465.348 | 1.2331 | Nutty/Fruity |
| 20 | 2-Ethyl-5-methylpyrazine | C13360640 | C_7_H_10_N_2_ | 122.2 | 1013.3 | 375.827 | 1.20811 | Nutty |
| 21 | 2,4,5-trimethylthiazole | C13623115 | C_6_H_9_NS | 127.2 | 1011.8 | 373.568 | 1.11703 | Chocolate |
| 22 | 4-Methylthiazole | C693958 | C_4_H_5_NS | 99.2 | 841.8 | 238.195 | 1.36227 | Green |
| 23 | 2-Ethylfuran | C3208160 | C_6_H_8_O | 96.1 | 693.9 | 162.1 | 1.30512 | Bready |
| Others | | | | | | | | |
| 24 | Acrylonitrile | C107131 | C_3_H_3_N | 53.1 | 532.3 | 113.172 | 1.09829 | Sulfurous |
| 25 | 3-Methylpentane | C96140 | C_6_H_14_ | 86.2 | 584.1 | 128.623 | 1.28729 | Spicy |
| 26 | 1-Pentene | C109671 | C_5_H_10_ | 70.1 | 458.3 | 91.068 | 1.25519 | Odour |
| 27 | 1,2-Dimethoxyethane | C110714 | C_4_H_10_O_2_ | 90.1 | 625.8 | 141.07 | 1.28373 | Ethereal |

Note: ^1)^ The molecular weights of the volatile compounds. ^2)^ The retention indexes of the volatile compounds in the GC column. ^3)^ The retention times in the capillary GC column was determined. ^4)^ The drift times in the drift tube. The number of observations for each mean value was five (n = 5).

**Supplemental Table 4**

Comparison of 12 characteristic volatile compounds in the roots, bulbs and flowers of *Allium mongolicum* Regel.

| No. | Compound | VIP | Peak intensity | | |
| --- | --- | --- | --- | --- | --- |
|  |  |  | Flowers | Roots | Bulbs |
| 1 | (E)-2-Octenal | 1.32 | 519.65^a^ | 665.49^a^ | 192.39^b^ |
| 2 | 6-Methyl-5-hepten-2-one | 1.67 | 1851.18^A^ | 1591.52^B^ | 884.64^C^ |
| 3 | 1-Pentanol | 1.51 | 312.46^C^ | 520.25^B^ | 861.12^A^ |
| 4 | 3-Methyl-2-butanol | 4.24 | 1940.89^A^ | 863.98^B^ | 1910.28^A^ |
| 5 | 2-Methyl-1-pentanol | 2.58 | 2144.87^A^ | 1973.13^B^ | 793.54^C^ |
| 6 | Linalyl acetate | 2.02 | 910.03^B^ | 1340.68^A^ | 569.42^C^ |
| 7 | Isobutyl butyrate | 1.53 | 382.86^B^ | 447.27^B^ | 808.12^A^ |
| 8 | Methyl butanoate | 1.37 | 761.75^A^ | 258.39^C^ | 605.74^B^ |
| 9 | Isopentyl formate | 1.22 | 262.45^B^ | 251.92^B^ | 555.46^A^ |
| 10 | 2,3-Dimethylpyrazine | 1.38 | 1423.92^A^ | 878.74^B^ | 353.84^C^ |
| 11 | 2,4,5-Trimethylthiazole | 1.77 | 2909.87^A^ | 2654.69^A^ | 1813.76^B^ |
| 12 | Acetophenone | 1.09 | 371.08^B^ | 239.28^C^ | 512.40^A^ |

Note: ^ab^ means within a row followed by different lowercase letters differ significantly (*P* < 0.05); ^ABC^ means in the same row with different capital letters indicate highly significant differences (*P* < 0.01). A total of 12 characteristic volatile compounds were selected based on the following screening conditions: VIP≥1 and *P*<0.05. The number of observations for each mean value was five (n = 5).

**Supplemental Table 5**

Identification of 47 volatile compounds in basal diet without additives and dietary AMRP supplementation of Angus calves in the right *longissimus thoracis* by gas chromatography–ion mobility spectrometry (GC–IMS).

| No. | Compound | CAS | Molecule formula | MW^1)^ | RI^2)^ | RT/s^3)^ | DT/s^4)^ | Odour description |
| --- | --- | --- | --- | --- | --- | --- | --- | --- |
| Aldehydes | | | | | | | | |
| 1 | 2-Methyl-2-propenal | C78853 | C_4_H_6_O | 70.1 | 572.3 | 128.871 | 1.22727 | Spicy |
| 2 | 2-Methyl-2-pentenal-M | C623369 | C_6_H_10_O | 98.1 | 826.6 | 225.764 | 1.16079 | Green/Fruity |
| 3 | 2-Methyl-2-pentenal-D | C623369 | C_6_H_10_O | 98.1 | 847.7 | 238.337 | 1.16284 | Spicy |
| 4 | 5-Methylfurfural | C620020 | C_6_H_6_O_2_ | 110.1 | 966.3 | 339.615 | 1.49268 | Caramel |
| 5 | 3-methylbutanal | C590863 | C_5_H_10_O | 86.1 | 614.9 | 141.722 | 1.20454 | Floral/Fruity |
| 6 | 2-Methylbutanal-D | C96173 | C_5_H_10_O | 86.1 | 685.3 | 162.94 | 1.41172 | Spicy |
| 7 | 2-Methylbutanal-M | C96173 | C_5_H_10_O | 86.1 | 669.2 | 158.087 | 1.41163 | Spicy |
| Ketones | | | | | | | | |
| 8 | 2,3-Butanedione-D | C431038 | C_4_H_6_O_2_ | 86.1 | 566.9 | 127.243 | 1.17385 | Buttery |
| 9 | 1-Penten-3-one-M | C1629589 | C_5_H_8_O | 84.1 | 663.7 | 156.437 | 1.08292 | Spicy/Onion |
| 10 | 1-Penten-3-one-D | C1629589 | C_5_H_8_O | 84.1 | 682 | 161.965 | 1.07791 | Spicy/Onion |
| 11 | 3-Pentanone | C96220 | C_5_H_10_O | 86.1 | 690.6 | 164.717 | 1.36816 | Creamy |
| 12 | 2,3-Butanedione-M | C431038 | C_4_H_6_O_2_ | 86.1 | 528.9 | 115.764 | 1.15327 | Balsamic |
| 13 | Cyclohexen-2-one-M | C930687 | C_6_H_8_O | 96.1 | 869.7 | 251.403 | 1.42027 | Citrus |
| 14 | 3-hydroxy-2-butanone-D | C513860 | C_4_H_8_O_2_ | 88.1 | 751.4 | 187.985 | 1.32746 | Sweet |
| 15 | 4-Methyl-3-penten-2-one | C141797 | C_6_H_10_O | 98.1 | 779.1 | 198.571 | 1.44284 | Sweet |
| 16 | 3-methyl-2-pentanone | C565617 | C_6_H_12_O | 100.2 | 749.2 | 187.146 | 1.47435 | Cheesy |
| 17 | Cyclohexen-2-one-D | C930687 | C_6_H_8_O | 96.1 | 902.1 | 274.791 | 1.42201 | Ethereal |
| 18 | 3-hydroxy-2-butanone-M | C513860 | C_4_H_8_O_2_ | 88.1 | 680.2 | 161.424 | 1.32975 | Balsamic |
| Alcohols | | | | | | | | |
| 19 | Hydroxyacetone-M | C116096 | C_3_H_6_O_2_ | 74.1 | 616.8 | 142.293 | 1.04389 | Spicy |
| 20 | Hydroxyacetone-D | C116096 | C_3_H_6_O_2_ | 74.1 | 655 | 153.812 | 1.04725 | Spicy |
| 21 | 2-Methylpropanol | C78831 | C_4_H_10_O | 74.1 | 609 | 139.935 | 1.36343 | Spicy |
| 22 | 3-methyl-1-butanol | C123513 | C_5_H_12_O | 88.1 | 704.1 | 169.884 | 1.47834 | Alcoholic |
| 23 | 1-Propanethiol | C107039 | C_3_H_8_S | 76.2 | 595.6 | 135.892 | 1.18291 | Odour |
| 24 | 1-pentanol | C71410 | C_5_H_12_O | 88.1 | 779.8 | 198.831 | 1.51768 | Green |
| 25 | 2-Methyl-1-butanol | C137326 | C_5_H_12_O | 88.1 | 767.2 | 194.027 | 1.4796 | Floral/Fruity |
| Esters | | | | | | | | |
| 26 | Ethyl formate | C109944 | C_3_H_6_O_2_ | 74.1 | 530.7 | 116.323 | 1.06067 | Floral |
| 27 | Propyl acetate | C109604 | C_5_H_10_O_2_ | 102.1 | 673 | 159.248 | 1.17161 | Fruity |
| 28 | Methyl acetate | C79209 | C_3_H_6_O_2_ | 74.1 | 523.1 | 114.026 | 1.17951 | Herbal |
| 29 | Ethyl propanoate | C105373 | C_5_H_10_O_2_ | 102.1 | 693.7 | 165.906 | 1.44519 | Fruity |
| 30 | ethyl acetate | C141786 | C_4_H_8_O_2_ | 88.1 | 631.5 | 146.736 | 1.32482 | Fruity/Alcoholic |
| Acids | | | | | | | | |
| 31 | Propanoic acid-D | C79094 | C_3_H_6_O_2_ | 74.1 | 694.6 | 166.243 | 1.28881 | Spicy |
| 32 | Propanoic acid-M | C79094 | C_3_H_6_O_2_ | 74.1 | 665.5 | 156.981 | 1.25746 | Spicy |
| 33 | 2-Methylpropanoic acid | C79312 | C_4_H_8_O_2_ | 88.1 | 771.9 | 195.817 | 1.15538 | Cheesy |
| 34 | Acetic acid | C64197 | C_2_H_4_O_2_ | 60.1 | 573.8 | 129.327 | 1.06442 | Pungent/Acidic |
| Heterocycles | | | | | | | | |
| 35 | 2-Butylfuran | C4466244 | C_8_H_12_O | 124.2 | 901.5 | 274.181 | 1.17945 | Pungent |
| 36 | Tetrahydro furane | C109999 | C_4_H_8_O | 72.1 | 632.4 | 146.996 | 1.22796 | Ethereal |
| 37 | 2,5-Dimethylfuran | C625865 | C_6_H_8_O | 96.1 | 678.9 | 161.005 | 1.36816 | Pungent |
| 38 | 2-Methylpyrazine | C109080 | C_5_H_6_N_2_ | 94.1 | 826.3 | 225.603 | 1.40315 | Nutty |
| Amine | | | | | | | | |
| 39 | 1-Propanamine | C107108 | C_3_H_9_N | 59.1 | 526.9 | 115.183 | 1.24268 | Ammonia |
| 40 | Ethanamine -D | C75047 | C_2_H_7_N | 45.1 | 384.1 | 72.093 | 1.09306 | Ammonia |
| 41 | Dimethylamine-M | C124403 | C_2_H_7_N | 45.1 | 412.8 | 80.744 | 0.92567 | Ammonia |
| 42 | Ethanamine-M | C75047 | C_2_H_7_N | 45.1 | 384.1 | 72.093 | 1.09306 | Ammonia |
| 43 | Dimethylamine-D | C124403 | C_2_H_7_N | 45.1 | 432.5 | 86.702 | 1.05019 | Ammonia |
| Other categories | | | | | | | | |
| 44 | Undecane | C1120214 | C_11_H_24_ | 156.3 | 162.1 | 5.124 | 1.04831 | — |
| 45 | N-Phenyl carbazole | C1150625 | C_18_H_13_N | 243.3 | 380.2 | 70.913 | 1.04631 | — |
| 46 | Acrylonitrile | C107131 | C_3_H_3_N | 53.1 | 474.8 | 99.446 | 1.10911 | Spicy/Onion |
| 47 | 1,2-Dimethoxyethane | C110714 | C_4_H_10_O_2_ | 90.1 | 611.1 | 140.57 | 1.117 | Aldehydic |

Note: M: monomer, D: dimer. ^1)^ The molecular weights of the volatile compounds. ^2)^ The retention indexes of the volatile compounds in the GC column. ^3)^ The retention times in the capillary GC column. ^4)^ The drift times in the drift tube. The number of observations for each mean value was six (n = 6).

**Supplemental Table 6**

A total of 199 differential lipid metabolites of the *longissimus thoracis* were identified in groups C and H in positive and negative ion modes, 112 of which were upregulated and 87 of which were downregulated.

| No. | Index | C_mean | H_mean | Log2_FC(H_mean/C_mean) | *P* value | VIP | Compounds | Class | IonFormula | RT | Up or  down |
| --- | --- | --- | --- | --- | --- | --- | --- | --- | --- | --- | --- |
| 1 | POS1452 | 41946477.98 | 144876379.2 | 1.788 | 6.97E-08 | 7.679 | TG(18:0_17:0_18:1) | TG | C56 H110 O6 N1 | 15.961 | Up |
| 2 | POS1238 | 54520994.38 | 139177211.7 | 1.352 | 8.27E-10 | 7.017 | TG(16:0_14:0_18:1) | TG | C51 H100 O6 N1 | 14.799 | Up |
| 3 | POS1250 | 39071530.72 | 123949088.9 | 1.666 | 4.29E-10 | 6.996 | TG(16:0_14:0_18:2) | TG | C51 H98 O6 N1 | 14.523 | Up |
| 4 | POS1419 | 66792628.03 | 151071290.1 | 1.177 | 8.17E-09 | 6.970 | TG(16:1_18:1_18:1) | TG | C55 H104 O6 N1 | 14.845 | Up |
| 5 | POS1343 | 31403358.4 | 114627514.3 | 1.868 | 9.14E-10 | 6.919 | TG(16:0_17:0_18:1) | TG | C54 H106 O6 N1 | 15.532 | Up |
| 6 | POS1248 | 36932771.88 | 112053991.9 | 1.601 | 5.83E-08 | 6.532 | TG(16:0_14:1_18:1) | TG | C51 H98 O6 N1 | 14.057 | Up |
| 7 | POS1270 | 12728757.44 | 84322878.04 | 2.728 | 3.37E-05 | 6.201 | TG(15:0_16:0_18:1) | TG | C52 H102 O6 N1 | 15.003 | Up |
| 8 | POS1459 | 36403437.94 | 99641580.9 | 1.453 | 1.72E-08 | 6.001 | TG(17:0_18:1_18:1) | TG | C56 H108 O6 N1 | 15.522 | Up |
| 9 | POS1491 | 39762683.84 | 95965266.3 | 1.271 | 1.55E-06 | 5.641 | TG(18:0_18:0_18:1) | TG | C57 H112 O6 N1 | 16.221 | Up |
| 10 | POS1379 | 89484104.07 | 144334045.3 | 0.690 | 2.32E-06 | 5.556 | TG(18:0_16:0_18:1) | TG | C55 H108 O6 N1 | 15.840 | Up |
| 11 | POS1202 | 17270975.33 | 65502421.91 | 1.923 | 2.45E-10 | 5.270 | TG(16:0_14:0_16:1) | TG | C49 H96 O6 N1 | 14.068 | Up |
| 12 | POS1351 | 32151680.44 | 81703911.04 | 1.346 | 1.92E-06 | 5.241 | TG(16:0_17:1_18:1) | TG | C54 H104 O6 N1 | 15.082 | Up |
| 13 | POS1301 | 72642244.7 | 113373115.3 | 0.642 | 1.58E-06 | 4.801 | TG(16:0_16:0_18:1) | TG | C53 H104 O6 N1 | 15.381 | Up |
| 14 | POS1425 | 16540418.94 | 51803822.93 | 1.647 | 7.86E-11 | 4.515 | TG(16:0_18:1_18:3) | TG | C55 H102 O6 N1 | 14.261 | Up |
| 15 | POS1325 | 21433792.75 | 59257356.62 | 1.467 | 0.000288227 | 4.423 | TG(16:0_16:0_18:3) | TG | C53 H100 O6 N1 | 14.106 | Up |
| 16 | POS1204 | 17225829.25 | 60086427.03 | 1.802 | 0.005089553 | 4.315 | TG(16:0_14:0_16:1) | TG | C49 H96 O6 N1 | 14.495 | Up |
| 17 | POS1326 | 9883783.17 | 50963682.14 | 2.366 | 0.010401855 | 4.251 | TG(16:0_16:0_18:3) | TG | C53 H100 O6 N1 | 14.659 | Up |
| 18 | POS1097 | 79255276.66 | 107905973.2 | 0.445 | 4.25E-09 | 4.028 | SPH(d16:0) | SPH | C16 H36 O2 N1 | 1.829 | Up |
| 19 | POS1275 | 8230321.231 | 35748011.94 | 2.119 | 8.43E-07 | 3.894 | TG(16:0_16:1_17:1) | TG | C52 H100 O6 N1 | 14.443 | Up |
| 20 | POS1178 | 6058758.722 | 27886798.35 | 2.202 | 1.44E-08 | 3.520 | TG(16:0_10:0_18:1) | TG | C47 H92 O6 N1 | 13.217 | Up |
| 21 | POS1251 | 7901005.847 | 26084853.77 | 1.723 | 1.08E-05 | 3.173 | TG(16:1_14:1_18:1) | TG | C51 H96 O6 N1 | 13.282 | Up |
| 22 | POS1356 | 5614442.505 | 22606587.61 | 2.010 | 8.47E-11 | 3.127 | TG(16:1_17:1_18:1) | TG | C54 H102 O6 N1 | 14.503 | Up |
| 23 | POS1231 | 8577560.06 | 25213232.56 | 1.556 | 2.76E-08 | 3.095 | TG(15:0_16:0_17:0) | TG | C51 H102 O6 N1 | 14.551 | Up |
| 24 | POS1217 | 2664908.417 | 19186608.45 | 2.848 | 4.62E-07 | 3.070 | TG(15:0_16:0_16:0) | TG | C50 H100 O6 N1 | 15.032 | Up |
| 25 | POS1519 | 15195400.46 | 31587016.59 | 1.056 | 5.55E-07 | 3.028 | TG(16:0_18:1_20:3) | TG | C57 H106 O6 N1 | 14.929 | Up |
| 26 | POS1465 | 12277932.37 | 26788039.77 | 1.126 | 2.99E-07 | 2.879 | TG(18:1_17:1_18:1) | TG | C56 H106 O6 N1 | 15.062 | Up |
| 27 | POS1206 | 5466031.899 | 19580480.14 | 1.841 | 1.35E-08 | 2.833 | TG(16:0_14:1_16:1) | TG | C49 H94 O6 N1 | 13.201 | Up |
| 28 | POS1733 | 1612755.337 | 14612582.8 | 3.180 | 3.10E-09 | 2.733 | ZyE(35:6) | ZyE | C62 H104 O2 N1 | 12.770 | Up |
| 29 | POS1220 | 2405189.047 | 14394224.26 | 2.581 | 2.34E-09 | 2.621 | TG(15:0_14:0_18:1) | TG | C50 H98 O6 N1 | 14.334 | Up |
| 30 | POS1485 | 8096761.844 | 19613032.74 | 1.276 | 9.51E-07 | 2.552 | TG(18:0_18:0_18:0) | TG | C57 H114 O6 N1 | 16.214 | Up |
| 31 | POS1545 | 5212268.779 | 16010744.66 | 1.619 | 2.90E-07 | 2.484 | TG(19:1_18:0_18:1) | TG | C58 H112 O6 N1 | 15.977 | Up |
| 32 | POS1488 | 13700356.59 | 24477831.37 | 0.837 | 3.25E-06 | 2.455 | TG(18:0_18:0_18:1) | TG | C57 H112 O6 N1 | 15.795 | Up |
| 33 | POS595 | 2204351.876 | 16531521.64 | 2.907 | 0.049066233 | 2.287 | PC(34:1e) | PC | C42 H85 O7 N1 P1 | 7.221 | Up |
| 34 | POS1264 | 4460711.515 | 13411062.38 | 1.588 | 2.20E-08 | 2.271 | TG(18:0_15:0_16:0) | TG | C52 H104 O6 N1 | 15.552 | Up |
| 35 | POS1540 | 2316938.598 | 10479669.52 | 2.177 | 1.45E-07 | 2.166 | TG(18:0_18:1_19:0) | TG | C58 H114 O6 N1 | 16.308 | Up |
| 36 | POS1224 | 1198649.73 | 8797189.082 | 2.876 | 7.09E-12 | 2.097 | TG(15:0_16:1_16:1) | TG | C50 H96 O6 N1 | 13.641 | Up |
| 37 | POS1328 | 2463114.99 | 9417832.169 | 1.935 | 1.57E-07 | 1.960 | TG(16:1_16:1_18:2) | TG | C53 H98 O6 N1 | 13.417 | Up |
| 38 | POS1162 | 2029467.992 | 8130874.381 | 2.002 | 2.83E-09 | 1.877 | TG(16:0_8:0_18:1) | TG | C45 H88 O6 N1 | 12.302 | Up |
| 39 | POS1720 | 680804.7846 | 6324807.323 | 3.216 | 6.07E-09 | 1.793 | ZyE(33:6) | ZyE | C60 H100 O2 N1 | 12.190 | Up |
| 40 | POS190 | 3620968.682 | 8763136.605 | 1.275 | 9.48E-09 | 1.726 | DG(30:0e) | DG | C33 H66 O4 Na1 | 14.795 | Up |
| 41 | POS256 | 3678965.956 | 8812873 | 1.260 | 1.48E-08 | 1.723 | DG(32:3e) | DG | C35 H65 O4 | 14.781 | Up |
| 42 | POS1526 | 3476850.384 | 9777613.422 | 1.492 | 0.00068386 | 1.706 | TG(18:1_18:1_18:3) | TG | C57 H104 O6 N1 | 14.291 | Up |
| 43 | POS1198 | 1371276.166 | 6483740.648 | 2.241 | 2.14E-07 | 1.699 | TG(16:0_14:0_16:0) | TG | C49 H98 O6 N1 | 14.698 | Up |
| 44 | POS1469 | 1688364.311 | 6729288.135 | 1.995 | 1.61E-09 | 1.689 | TG(18:1_17:1_18:2) | TG | C56 H104 O6 N1 | 14.596 | Up |
| 45 | POS1188 | 701206.0166 | 5681827.527 | 3.018 | 2.45E-07 | 1.685 | TG(15:0_14:0_16:0) | TG | C48 H96 O6 N1 | 14.344 | Up |
| 46 | POS1574 | 2766102.828 | 7670308.144 | 1.471 | 6.10E-11 | 1.683 | TG(18:0_18:1_20:1) | TG | C59 H114 O6 N1 | 16.180 | Up |
| 47 | POS1595 | 1981598.38 | 6910926.704 | 1.802 | 6.53E-10 | 1.674 | TG(18:1_18:1_20:4) | TG | C59 H106 O6 N1 | 14.532 | Up |
| 48 | POS1587 | 2673907.123 | 7368681.85 | 1.462 | 1.18E-07 | 1.645 | TG(18:1_18:1_20:3) | TG | C59 H108 O6 N1 | 14.976 | Up |
| 49 | POS233 | 8218054.202 | 12952363.52 | 0.656 | 6.62E-07 | 1.635 | DG(32:0e) | DG | C35 H70 O4 Na1 | 15.375 | Up |
| 50 | POS1568 | 1825785.422 | 6813064.731 | 1.900 | 5.94E-05 | 1.630 | TG(18:0_18:1_20:0) | TG | C59 H116 O6 N1 | 16.471 | Up |
| 51 | POS542 | 36175012.3 | 41240761.23 | 0.189 | 0.000206873 | 1.623 | PC(32:1) | PC | C40 H79 O8 N1 P1 | 6.094 | Up |
| 52 | POS1735 | 1144112.369 | 6042067.087 | 2.401 | 6.11E-06 | 1.619 | ZyE(35:6) | ZyE | C62 H104 O2 N1 | 13.141 | Up |
| 53 | POS1598 | 621641.8322 | 5004639.948 | 3.009 | 5.91E-06 | 1.550 | TG(20:4e_18:1_18:1) | TG | C59 H105 O5 | 13.146 | Up |
| 54 | POS1597 | 1036885.154 | 5367279.306 | 2.372 | 2.00E-05 | 1.526 | TG(20:3e_18:1_18:2) | TG | C59 H105 O5 | 12.759 | Up |
| 55 | POS1417 | 2878734.617 | 7056824.678 | 1.294 | 2.13E-05 | 1.513 | TG(16:0_18:1_18:2) | TG | C55 H104 O6 N1 | 14.268 | Up |
| 56 | POS1353 | 1349270.639 | 5237844.153 | 1.957 | 8.34E-09 | 1.492 | TG(16:0_17:1_18:1) | TG | C54 H104 O6 N1 | 15.555 | Up |
| 57 | POS1516 | 787678.7667 | 4733429.072 | 2.587 | 4.43E-05 | 1.459 | TG(18:0e_18:1_18:2) | TG | C57 H106 O5 Na1 | 12.746 | Up |
| 58 | POS1550 | 2932396.176 | 6694914.918 | 1.191 | 8.37E-08 | 1.458 | TG(19:1_18:1_18:1) | TG | C58 H110 O6 N1 | 15.647 | Up |
| 59 | POS1180 | 2412184.329 | 6025586.45 | 1.321 | 1.84E-06 | 1.431 | TG(16:0_10:1_18:1) | TG | C47 H90 O6 N1 | 12.338 | Up |
| 60 | POS692 | 32563028.95 | 36715600.43 | 0.173 | 0.00333583 | 1.392 | PC(36:4) | PC | C44 H81 O8 N1 P1 | 5.999 | Up |
| 61 | POS274 | 1121456.246 | 4456324.029 | 1.990 | 1.48E-09 | 1.390 | DG(33:3e) | DG | C36 H67 O4 | 15.114 | Up |
| 62 | POS211 | 1121497.385 | 4454717.785 | 1.990 | 1.46E-09 | 1.389 | DG(31:0e) | DG | C34 H68 O4 Na1 | 15.008 | Up |
| 63 | POS1278 | 1155572.856 | 4581858.126 | 1.987 | 1.90E-06 | 1.380 | TG(15:0_16:0_18:3) | TG | C52 H98 O6 N1 | 13.692 | Up |
| 64 | POS262 | 1359747.369 | 4718341.088 | 1.795 | 1.22E-07 | 1.377 | DG(33:0e) | DG | C36 H72 O4 Na1 | 15.518 | Up |
| 65 | POS1337 | 2959110.51 | 6316952.686 | 1.094 | 2.78E-06 | 1.371 | TG(18:0_16:0_17:0) | TG | C54 H108 O6 N1 | 15.983 | Up |
| 66 | POS327 | 1443854.304 | 4718969.092 | 1.709 | 2.16E-08 | 1.365 | DG(35:3e) | DG | C38 H71 O4 | 15.669 | Up |
| 67 | POS1591 | 477862.7959 | 3789302.419 | 2.987 | 1.97E-06 | 1.363 | TG(18:3e_18:1_20:1) | TG | C59 H107 O5 | 12.710 | Up |
| 68 | POS1147 | 802349.4089 | 3845727.717 | 2.261 | 9.72E-08 | 1.309 | TG(16:0_10:0_14:0) | TG | C43 H86 O6 N1 | 12.276 | Up |
| 69 | POS292 | 482381.5949 | 3446724.627 | 2.837 | 2.91E-10 | 1.302 | DG(34:1e) | DG | C37 H72 O4 Na1 | 13.115 | Up |
| 70 | POS1427 | 572977.088 | 3556125.25 | 2.634 | 2.67E-09 | 1.300 | TG(16:0_16:0_20:4) | TG | C55 H102 O6 N1 | 14.662 | Up |
| 71 | POS201 | 1706528.717 | 4518139.12 | 1.405 | 7.02E-09 | 1.276 | DG(30:2e) | DG | C33 H63 O4 | 14.800 | Up |
| 72 | POS1602 | 303951.0072 | 3134911.356 | 3.367 | 2.50E-08 | 1.269 | TG(14:1e_20:0_22:6) | TG | C59 H103 O5 | 12.406 | Up |
| 73 | POS1359 | 656921.7653 | 3350848.592 | 2.351 | 2.54E-11 | 1.243 | TG(15:0_18:1_18:3) | TG | C54 H100 O6 N1 | 13.850 | Up |
| 74 | POS1433 | 1399087.408 | 3927739.132 | 1.489 | 2.73E-08 | 1.207 | TG(18:4_16:0_18:1) | TG | C55 H100 O6 N1 | 13.510 | Up |
| 75 | POS217 | 540412.7825 | 3027681.426 | 2.486 | 8.30E-10 | 1.201 | DG(31:2e) | DG | C34 H65 O4 | 15.014 | Up |
| 76 | POS241 | 2618392.587 | 5042655.663 | 0.946 | 9.57E-08 | 1.183 | DG(32:1e) | DG | C35 H68 O4 Na1 | 14.766 | Up |
| 77 | POS1461 | 878331.4148 | 3279501.987 | 1.901 | 4.25E-07 | 1.168 | TG(18:0_17:1_18:1) | TG | C56 H108 O6 N1 | 15.992 | Up |
| 78 | POS498 | 427987.9969 | 2800278.268 | 2.710 | 6.34E-08 | 1.164 | MG(32:0) | MG | C35 H70 O4 Na1 | 13.123 | Up |
| 79 | POS1601 | 420590.7935 | 3098153.795 | 2.881 | 0.000220669 | 1.149 | TG(20:3e_18:2_18:2) | TG | C59 H103 O5 | 12.001 | Up |
| 80 | POS1722 | 548331.8118 | 2962734.377 | 2.434 | 5.75E-06 | 1.149 | ZyE(33:6) | ZyE | C60 H100 O2 N1 | 12.629 | Up |
| 81 | POS296 | 4370090.526 | 6655607.538 | 0.607 | 3.42E-08 | 1.143 | DG(34:1e) | DG | C37 H72 O4 Na1 | 15.313 | Up |
| 82 | POS1592 | 352708.9133 | 2567203.178 | 2.864 | 3.49E-08 | 1.123 | TG(20:0e_18:1_18:4) | TG | C59 H107 O5 | 13.162 | Up |
| 83 | POS1746 | 203707.6777 | 2404852.553 | 3.561 | 9.41E-09 | 1.123 | ZyE(37:6) | ZyE | C64 H108 O2 N1 | 13.896 | Up |
| 84 | POS1190 | 342026.3383 | 2543007.81 | 2.894 | 2.49E-07 | 1.120 | TG(15:0_14:0_16:1) | TG | C48 H94 O6 N1 | 13.505 | Up |
| 85 | POS1104 | 2698602.947 | 4811015.008 | 0.834 | 3.85E-12 | 1.106 | SPH(d22:0) | SPH | C22 H48 O2 N1 | 1.706 | Up |
| 86 | POS270 | 760943.9932 | 2831405.643 | 1.896 | 5.55E-10 | 1.091 | DG(33:2e) | DG | C36 H69 O4 | 15.539 | Up |
| 87 | POS253 | 280143.0433 | 2290666.498 | 3.032 | 1.83E-10 | 1.075 | DG(32:3e) | DG | C35 H65 O4 | 12.415 | Up |
| 88 | POS1330 | 419343.9722 | 2373359.341 | 2.501 | 6.54E-08 | 1.056 | TG(18:0_14:3_18:1) | TG | C53 H98 O6 N1 | 13.843 | Up |
| 89 | POS1177 | 370801.6109 | 2201178.991 | 2.570 | 5.47E-09 | 1.029 | TG(16:0_14:0_14:0) | TG | C47 H94 O6 N1 | 13.946 | Up |
| 90 | POS1209 | 820526.3807 | 2604307.601 | 1.666 | 2.30E-10 | 1.016 | TG(16:1_14:1_16:1) | TG | C49 H92 O6 N1 | 12.437 | Up |
| 91 | POS307 | 1828576.179 | 3674532.917 | 1.007 | 7.38E-06 | 1.013 | DG(34:2e) | DG | C37 H71 O4 | 15.844 | Up |
| 92 | POS1400 | 189356967.3 | 45208255.88 | -2.066 | 0.009942443 | 7.766 | TG(16:0_18:1_18:1) | TG | C55 H106 O6 N1 | 16.893 | Down |
| 93 | POS606 | 133425347.8 | 97279583.49 | -0.456 | 7.20E-06 | 4.401 | PC(16:2e_18:0) | PC | C42 H83 O7 N1 P1 | 7.653 | Down |
| 94 | POS1514 | 115888799.4 | 88128611.71 | -0.395 | 0.00286544 | 3.485 | TG(18:1_18:1_18:1) | TG | C57 H108 O6 N1 | 15.271 | Down |
| 95 | POS677 | 64534660.27 | 44672159.8 | -0.531 | 2.24E-06 | 3.287 | PC(36:2) | PC | C44 H85 O8 N1 P1 | 7.361 | Down |
| 96 | POS539 | 106487849.8 | 86994610.12 | -0.292 | 0.000636878 | 3.049 | PC(16:0e_16:0) | PC | C40 H82 O7 N1 P1 Na1 | 6.729 | Down |
| 97 | POS897 | 44412173.63 | 26272109.76 | -0.757 | 0.001167465 | 2.918 | PE(14:0e_23:0) | PE | C42 H86 O7 N1 P1 Na1 | 7.859 | Down |
| 98 | POS672 | 53083404.97 | 40826927.34 | -0.379 | 0.001710552 | 2.368 | PC(36:1) | PC | C44 H87 O8 N1 P1 | 8.234 | Down |
| 99 | POS605 | 30461373.56 | 21154819.2 | -0.526 | 2.10E-07 | 2.284 | PC(34:2e) | PC | C42 H83 O7 N1 P1 | 6.932 | Down |
| 100 | POS643 | 16494389.81 | 11078291.74 | -0.574 | 1.00E-06 | 1.711 | PC(35:2e) | PC | C43 H85 O7 N1 P1 | 8.001 | Down |
| 101 | POS674 | 8186225.525 | 3629906.098 | -1.173 | 4.67E-08 | 1.600 | PC(18:0e_18:1) | PC | C44 H89 O7 N1 P1 | 8.920 | Down |
| 102 | POS11 | 6040629.669 | 1727932.897 | -1.806 | 1.22E-16 | 1.581 | AcCa(18:1) | AcCa | C25 H48 O4 N1 | 1.363 | Down |
| 103 | POS679 | 10367013.54 | 5221584.16 | -0.989 | 0.021573495 | 1.389 | PC(36:2e) | PC | C44 H87 O7 N1 P1 | 7.852 | Down |
| 104 | POS883 | 10262191.94 | 6709531.275 | -0.613 | 1.18E-05 | 1.372 | PE(18:0p_18:2) | PE | C41 H79 O7 N1 P1 | 8.225 | Down |
| 105 | POS565 | 9743275.008 | 6886328.29 | -0.501 | 1.98E-07 | 1.268 | PC(11:0_22:1) | PC | C41 H80 O8 N1 P1 Na1 | 7.378 | Down |
| 106 | POS574 | 5328451.117 | 2674127.313 | -0.995 | 2.24E-07 | 1.214 | PC(33:2e) | PC | C41 H81 O7 N1 P1 | 6.960 | Down |
| 107 | POS929 | 10627549.29 | 8007325.064 | -0.408 | 5.26E-06 | 1.193 | PE(18:0_20:4) | PE | C43 H79 O8 N1 P1 | 7.436 | Down |
| 108 | POS658 | 9135359.179 | 6610986.021 | -0.467 | 1.30E-07 | 1.190 | PC(35:4) | PC | C43 H79 O8 N1 P1 | 7.397 | Down |
| 109 | POS874 | 9341565.173 | 6893105.172 | -0.439 | 9.05E-07 | 1.170 | PE(18:0_18:1) | PE | C41 H80 O8 N1 P1 Na1 | 7.425 | Down |
| 110 | POS480 | 4973328.731 | 2771842.901 | -0.843 | 7.59E-13 | 1.126 | LPE(18:0) | LPE | C23 H49 O7 N1 P1 | 1.863 | Down |
| 111 | POS631 | 8954800.115 | 6623117.678 | -0.435 | 0.000103248 | 1.088 | PC(26:1_9:0) | PC | C43 H85 O8 N1 P1 | 7.530 | Down |
| 112 | POS473 | 3832851.597 | 1499243.736 | -1.354 | 0.000200017 | 1.061 | LPC(36:1) | LPC | C44 H88 O7 N1 P1 Na1 | 7.466 | Down |
| 113 | POS822 | 4174716.91 | 2237596.269 | -0.900 | 3.27E-13 | 1.056 | PE(12:0e_6:0) | PE | C23 H49 O7 N1 P1 | 1.859 | Down |
| 114 | POS653 | 10644708.99 | 8643455.684 | -0.300 | 1.33E-05 | 1.036 | PC(35:3e) | PC | C43 H83 O7 N1 P1 | 7.099 | Down |
| 115 | NEG289 | 59186.71303 | 6105490.469 | 6.689 | 0.003076124 | 6.689 | PC(16:0_18:2) | PC | C43 H81 O10 N1 P1 | 7.678 | Up |
| 116 | NEG587 | 3056657.193 | 6686454.92 | 1.129 | 7.83E-09 | 1.129 | PI(18:0_20:4)-H | PI | C47 H82 O13 N0 P1 | 7.129 | Up |
| 117 | NEG477 | 1795146.305 | 5388928.27 | 1.586 | 1.14E-05 | 1.586 | PE(36:5e)-H | PE | C41 H73 O7 N1 P1 | 8.244 | Up |
| 118 | NEG573 | 1874605.286 | 4329119.03 | 1.207 | 7.03E-11 | 1.207 | PI(18:0_18:1)-H | PI | C45 H84 O13 N0 P1 | 8.268 | Up |
| 119 | NEG345 | 515307.2028 | 2457258.158 | 2.254 | 0.007878904 | 2.254 | PC(36:3e) | PC | C45 H85 O9 N1 P1 | 9.277 | Up |
| 120 | NEG476 | 5887372.026 | 7222383.559 | 0.295 | 1.58E-08 | 0.295 | PE(16:1e_20:4)-H | PE | C41 H73 O7 N1 P1 | 7.759 | Up |
| 121 | NEG611 | 1352417.025 | 2749649.45 | 1.024 | 0.000123392 | 1.024 | PS(18:0_18:1)-H | PS | C42 H79 O10 N1 P1 | 9.964 | Up |
| 122 | NEG726 | 852649.4092 | 2065474.36 | 1.276 | 7.72E-11 | 1.276 | ST(m42:6)-H | ST | C48 H82 O10 N1 S1 | 8.228 | Up |
| 123 | NEG501 | 11273371.85 | 12560864.78 | 0.156 | 0.000220645 | 0.156 | PE(18:1e_20:4)-H | PE | C43 H77 O7 N1 P1 | 8.913 | Up |
| 124 | NEG307 | 5429365.571 | 6606863.243 | 0.283 | 2.60E-06 | 0.283 | PC(34:5e)-CH3 | PC | C41 H73 O7 N1 P1 | 7.810 | Up |
| 125 | NEG506 | 3509076.721 | 4473447.461 | 0.350 | 1.78E-06 | 0.350 | PE(18:1e_20:5)-H | PE | C43 H75 O7 N1 P1 | 7.859 | Up |
| 126 | NEG598 | 548229.4843 | 1146021.165 | 1.064 | 1.46E-08 | 1.064 | PI(18:0_22:5)-H | PI | C49 H84 O13 N0 P1 | 7.126 | Up |
| 127 | NEG585 | 559956.992 | 1149242.592 | 1.037 | 3.05E-08 | 1.037 | PI(18:0_20:3)-H | PI | C47 H84 O13 N0 P1 | 7.799 | Up |
| 128 | NEG586 | 727594.8209 | 1278134.783 | 0.813 | 5.18E-08 | 0.813 | PI(18:0_20:3)-H | PI | C47 H84 O13 N0 P1 | 8.072 | Up |
| 129 | NEG362 | 4471277.31 | 5069712.941 | 0.181 | 0.000277603 | 0.181 | PC(16:1e_20:4) | PC | C45 H81 O9 N1 P1 | 7.439 | Up |
| 130 | NEG479 | 1114610.103 | 1646775.369 | 0.563 | 5.19E-05 | 0.563 | PE(16:1e_20:5)-H | PE | C41 H71 O7 N1 P1 | 7.001 | Up |
| 131 | NEG574 | 486505.5861 | 963274.1436 | 0.985 | 5.35E-09 | 0.985 | PI(18:0_18:2)-H | PI | C45 H82 O13 N0 P1 | 7.370 | Up |
| 132 | NEG584 | 249265.4534 | 558848.1937 | 1.165 | 6.13E-10 | 1.165 | PI(18:0_20:2)-H | PI | C47 H86 O13 N0 P1 | 8.383 | Up |
| 133 | NEG597 | 509580.6324 | 824384.5267 | 0.694 | 1.57E-07 | 0.694 | PI(18:0_22:4)-H | PI | C49 H86 O13 N0 P1 | 7.919 | Up |
| 134 | NEG366 | 1237219.1 | 1576831.973 | 0.350 | 0.004312263 | 0.350 | PC(16:1e_20:5) | PC | C45 H79 O9 N1 P1 | 6.630 | Up |
| 135 | NEG676 | 736957.2072 | 967518.3929 | 0.393 | 6.67E-06 | 0.393 | PS(40:7e)-H | PS | C46 H77 O9 N1 P1 | 8.991 | Up |
| 136 | NEG115 | 14301720.2 | 5164958.127 | -1.469 | 1.21E-06 | 6.998 | Hex1Cer(d36:1+O) | Hex1Cer | C43 H82 O11 N1 | 8.280 | Down |
| 137 | NEG197 | 10535591.69 | 4003116.884 | -1.396 | 2.80E-12 | 6.067 | LPE(18:0)-H | LPE | C23 H47 O7 N1 P1 | 2.134 | Down |
| 138 | NEG134 | 13916871.7 | 8808294.415 | -0.660 | 0.005305332 | 4.632 | Hex1Cer(t36:1) | Hex1Cer | C43 H82 O11 N1 | 8.316 | Down |
| 139 | NEG292 | 14122366.44 | 10439360.54 | -0.436 | 1.04E-07 | 4.534 | PC(16:1e_18:1) | PC | C43 H83 O9 N1 P1 | 8.490 | Down |
| 140 | NEG294 | 13054684.3 | 9642915.203 | -0.437 | 1.40E-07 | 4.352 | PC(16:1e_18:1) | PC | C43 H83 O9 N1 P1 | 8.932 | Down |
| 141 | NEG466 | 8900146.231 | 5841941.396 | -0.607 | 1.42E-09 | 4.136 | PE(18:1e_18:1)-H | PE | C41 H79 O7 N1 P1 | 10.227 | Down |
| 142 | NEG469 | 11905406.66 | 9005956.046 | -0.403 | 0.000371045 | 3.789 | PE(18:1e_18:2)-H | PE | C41 H77 O7 N1 P1 | 9.262 | Down |
| 143 | NEG285 | 4802108.688 | 2537572.58 | -0.920 | 7.79E-06 | 3.482 | PC(16:0e_18:1) | PC | C43 H85 O9 N1 P1 | 8.847 | Down |
| 144 | NEG721 | 3673939.834 | 658598.2179 | -2.480 | 0.041192708 | 3.220 | SPHP(m20:1) | SPHP | C21 H43 O6 N1 P1 | 2.091 | Down |
| 145 | NEG338 | 4786106.023 | 3108410.273 | -0.623 | 9.57E-09 | 3.062 | PC(18:1e_18:1) | PC | C45 H87 O9 N1 P1 | 9.780 | Down |
| 146 | NEG497 | 10084422.83 | 8409538.685 | -0.262 | 9.82E-09 | 3.048 | PE(18:0_20:4)-H | PE | C43 H77 O8 N1 P1 | 8.451 | Down |
| 147 | NEG351 | 10073445.67 | 8417315.22 | -0.259 | 1.70E-08 | 3.029 | PC(16:0_20:4)-CH3 | PC | C43 H77 O8 N1 P1 | 8.427 | Down |
| 148 | NEG447 | 5542849.514 | 3952712.938 | -0.488 | 1.80E-08 | 2.962 | PE(16:1e_18:1)-H | PE | C39 H75 O7 N1 P1 | 9.002 | Down |
| 149 | NEG332 | 2398494.483 | 888645.1235 | -1.432 | 3.83E-07 | 2.875 | PC(18:0e_18:1) | PC | C45 H89 O9 N1 P1 | 9.984 | Down |
| 150 | NEG344 | 4917670.573 | 2902817.818 | -0.761 | 0.007053577 | 2.846 | PC(18:2e_18:1) | PC | C45 H85 O9 N1 P1 | 8.786 | Down |
| 151 | NEG178 | 1555500.191 | 472238.7373 | -1.720 | 4.00E-14 | 2.473 | LPC(18:1) | LPC | C27 H53 O9 N1 P1 | 1.461 | Down |
| 152 | NEG290 | 3216765.19 | 2110530.789 | -0.608 | 3.63E-08 | 2.466 | PC(16:0e_18:2) | PC | C43 H83 O9 N1 P1 | 7.908 | Down |
| 153 | NEG462 | 1236820.476 | 178375.0601 | -2.794 | 7.61E-14 | 2.445 | PE(18:0e_18:1)-H | PE | C41 H81 O7 N1 P1 | 10.420 | Down |
| 154 | NEG133 | 3200589.619 | 2138216.311 | -0.582 | 9.56E-08 | 2.430 | Hex1Cer(t36:1) | Hex1Cer | C43 H82 O11 N1 | 7.900 | Down |
| 155 | NEG464 | 4734170.333 | 3544029.694 | -0.418 | 0.000358038 | 2.386 | PE(18:1_18:1)-H | PE | C41 H77 O8 N1 P1 | 8.686 | Down |
| 156 | NEG165 | 2066544.372 | 1053452.271 | -0.972 | 3.66E-11 | 2.385 | LPC(16:0) | LPC | C25 H51 O9 N1 P1 | 1.480 | Down |
| 157 | NEG301 | 10410564.34 | 9149629.012 | -0.186 | 0.004103139 | 2.314 | PC(16:1e_18:2) | PC | C43 H81 O9 N1 P1 | 7.702 | Down |
| 158 | NEG449 | 3299036.133 | 2280065.625 | -0.533 | 1.79E-05 | 2.286 | PE(16:1e_18:2)-H | PE | C39 H73 O7 N1 P1 | 8.018 | Down |
| 159 | NEG201 | 2885192.408 | 1954384.073 | -0.562 | 7.79E-10 | 2.283 | LPE(18:1e)-H | LPE | C23 H47 O6 N1 P1 | 2.434 | Down |
| 160 | NEG58 | 4280466.603 | 3284666.712 | -0.382 | 0.000429943 | 2.213 | Cer(d18:1_18:0) | Cer | C37 H72 O5 N1 | 9.497 | Down |
| 161 | NEG193 | 3333586.437 | 2457120.732 | -0.440 | 2.98E-06 | 2.172 | LPE(16:1e)-H | LPE | C21 H43 O6 N1 P1 | 1.760 | Down |
| 162 | NEG282 | 20261850.46 | 19346586.06 | -0.067 | 0.008419068 | 1.947 | PC(16:0_18:1) | PC | C43 H83 O10 N1 P1 | 8.141 | Down |
| 163 | NEG461 | 4078276.465 | 3378365.04 | -0.272 | 6.52E-05 | 1.900 | PE(18:0_18:1)-H | PE | C41 H79 O8 N1 P1 | 9.635 | Down |
| 164 | NEG199 | 939650.4316 | 309988.9014 | -1.600 | 1.50E-10 | 1.883 | LPE(18:1)-H | LPE | C23 H45 O7 N1 P1 | 1.561 | Down |
| 165 | NEG48 | 932448.7829 | 215883.1212 | -2.111 | 0.000920582 | 1.855 | CL(85:14)-2H | CL | C94 H154 O17 P2 | 6.780 | Down |
| 166 | NEG132 | 1049912.422 | 419907.8226 | -1.322 | 5.76E-06 | 1.831 | Hex1Cer(t35:1) | Hex1Cer | C42 H80 O11 N1 | 8.110 | Down |
| 167 | NEG715 | 1546220.434 | 958892.1424 | -0.689 | 1.90E-06 | 1.782 | SM(d42:2) | SM | C48 H94 O8 N2 P1 | 10.392 | Down |
| 168 | NEG696 | 2771022.648 | 2259137.237 | -0.295 | 1.64E-05 | 1.641 | SM(d34:1) | SM | C40 H80 O8 N2 P1 | 6.932 | Down |
| 169 | NEG288 | 11019456.03 | 10309244.35 | -0.096 | 0.025695773 | 1.633 | PC(16:1_18:1) | PC | C43 H81 O10 N1 P1 | 7.206 | Down |
| 170 | NEG170 | 1412640.556 | 937569.9516 | -0.591 | 8.71E-09 | 1.624 | LPC(16:1e) | LPC | C25 H51 O8 N1 P1 | 1.631 | Down |
| 171 | NEG330 | 5245601.063 | 4675700.82 | -0.166 | 0.001764735 | 1.613 | PC(18:0_18:1) | PC | C45 H87 O10 N1 P1 | 9.263 | Down |
| 172 | NEG498 | 4745280.26 | 4206627.228 | -0.174 | 0.002421585 | 1.564 | PE(18:1e_20:3)-H | PE | C43 H79 O7 N1 P1 | 9.572 | Down |
| 173 | NEG456 | 1031701.774 | 598609.2193 | -0.785 | 9.20E-10 | 1.559 | PE(18:1e_17:1)-H | PE | C40 H77 O7 N1 P1 | 9.655 | Down |
| 174 | NEG340 | 1762112.776 | 1333585.744 | -0.402 | 2.74E-08 | 1.544 | PC(18:1_18:2) | PC | C45 H83 O10 N1 P1 | 7.273 | Down |
| 175 | NEG77 | 1433621.612 | 997327.8996 | -0.524 | 0.00010783 | 1.499 | Cer(d18:1_24:1) | Cer | C43 H82 O5 N1 | 11.846 | Down |
| 176 | NEG353 | 1484125.705 | 906226.8522 | -0.712 | 0.014618115 | 1.491 | PC(16:0e_20:4) | PC | C45 H83 O9 N1 P1 | 7.656 | Down |
| 177 | NEG630 | 1640340.138 | 1187726.14 | -0.466 | 0.001523777 | 1.441 | PS(38:1e)-H | PS | C44 H85 O9 N1 P1 | 9.036 | Down |
| 178 | NEG473 | 2770562.15 | 2370489.666 | -0.225 | 0.001679626 | 1.356 | PE(18:2e_18:2)-H | PE | C41 H75 O7 N1 P1 | 8.333 | Down |
| 179 | NEG182 | 442840.2705 | 141244.5266 | -1.649 | 3.64E-15 | 1.304 | LPC(18:2) | LPC | C27 H51 O9 N1 P1 | 1.219 | Down |
| 180 | NEG89 | 1643071.102 | 1317885.71 | -0.318 | 0.000205939 | 1.270 | Cer(m18:1_23:0+O) | Cer | C42 H82 O5 N1 | 12.412 | Down |
| 181 | NEG202 | 371902.4676 | 88201.1785 | -2.076 | 2.24E-15 | 1.266 | LPE(18:2)-H | LPE | C23 H43 O7 N1 P1 | 1.269 | Down |
| 182 | NEG167 | 479531.0828 | 203872.2125 | -1.234 | 1.74E-12 | 1.246 | LPC(16:0e) | LPC | C25 H53 O8 N1 P1 | 1.713 | Down |
| 183 | NEG467 | 1290979.531 | 979289.6676 | -0.399 | 0.000341188 | 1.225 | PE(18:1_18:2)-H | PE | C41 H75 O8 N1 P1 | 7.629 | Down |
| 184 | NEG277 | 355691.2204 | 91198.77716 | -1.964 | 1.78E-12 | 1.222 | PC(16:1e_17:1) | PC | C42 H81 O9 N1 P1 | 7.931 | Down |
| 185 | NEG734 | 9901838.781 | 9514949.96 | -0.058 | 0.02339354 | 1.193 | phSM(t18:1_18:1) | phSM | C42 H82 O10 N2 P1 | 8.143 | Down |
| 186 | NEG643 | 1528441.941 | 1264758.161 | -0.273 | 7.47E-06 | 1.186 | PS(20:3e_18:1)-H | PS | C44 H79 O9 N1 P1 | 10.239 | Down |
| 187 | NEG337 | 923544.9155 | 679199.8349 | -0.443 | 1.08E-06 | 1.150 | PC(18:0e_18:2) | PC | C45 H87 O9 N1 P1 | 8.859 | Down |
| 188 | NEG279 | 445873.5491 | 214107.6061 | -1.058 | 3.79E-11 | 1.144 | PC(16:2e_17:1) | PC | C42 H79 O9 N1 P1 | 7.130 | Down |
| 189 | NEG175 | 536361.6974 | 310782.8959 | -0.787 | 3.50E-10 | 1.122 | LPC(18:0) | LPC | C27 H55 O9 N1 P1 | 1.997 | Down |
| 190 | NEG257 | 897983.5 | 583423.069 | -0.622 | 0.009682672 | 1.116 | PC(16:0_16:0) | PC | C41 H81 O10 N1 P1 | 7.958 | Down |
| 191 | NEG519 | 925905.065 | 714027.8996 | -0.375 | 2.19E-08 | 1.086 | PE(18:0_22:5)-H | PE | C45 H79 O8 N1 P1 | 8.428 | Down |
| 192 | NEG72 | 398196.3861 | 192081.4663 | -1.052 | 1.42E-05 | 1.041 | Cer(d18:2_23:0) | Cer | C42 H80 O5 N1 | 11.367 | Down |
| 193 | NEG312 | 1207000.458 | 1005930.239 | -0.263 | 1.46E-05 | 1.031 | PC(17:0_18:1) | PC | C44 H85 O10 N1 P1 | 8.521 | Down |
| 194 | NEG207 | 350569.1615 | 162729.4676 | -1.107 | 2.86E-12 | 1.028 | LPE(20:4)-H | LPE | C25 H43 O7 N1 P1 | 1.229 | Down |
| 195 | NEG266 | 765491.4247 | 574836.6345 | -0.413 | 4.03E-07 | 1.025 | PC(16:1e_16:1) | PC | C41 H79 O9 N1 P1 | 7.484 | Down |
| 196 | NEG59 | 499155.9104 | 308749.9322 | -0.693 | 4.72E-08 | 1.024 | Cer(d18:2_18:0) | Cer | C37 H70 O5 N1 | 8.424 | Down |
| 197 | NEG714 | 2484950.122 | 2249300.326 | -0.144 | 0.003302807 | 1.020 | SM(d42:1) | SM | C48 H96 O8 N2 P1 | 11.608 | Down |
| 198 | NEG470 | 442899.2745 | 159245.3698 | -1.476 | 0.031645659 | 1.005 | PE(18:1e_18:2)-H | PE | C41 H77 O7 N1 P1 | 9.702 | Down |
| 199 | NEG458 | 741395.6442 | 560394.3693 | -0.404 | 5.64E-08 | 1.005 | PE(18:2e_17:1)-H | PE | C40 H75 O7 N1 P1 | 8.508 | Down |

**Supplemental Table 7**

A total of 30 key lipid metabolites of the *longissimus thoracis* were identified in groups C and H in positive and negative ion modes, 18 of which were upregulated and 12 were downregulated

| No. | Index | C_mean | H_mean | Log2_FC(H_mean/C_mean) | *P* value | VIP | Compounds | Class | IonFormula | RT | Up or  down |
| --- | --- | --- | --- | --- | --- | --- | --- | --- | --- | --- | --- |
| 1 | POS1400 | 189356967.3 | 45208255.88 | -2.066 | 0.009942443 | 7.766 | TG(16:0_18:1_18:1) | TG | C55 H106 O6 N1 | 16.893 | Down |
| 2 | POS1452 | 41946477.98 | 144876379.2 | 1.788 | 6.97E-08 | 7.679 | TG(18:0_17:0_18:1) | TG | C56 H110 O6 N1 | 15.961 | Up |
| 3 | POS1238 | 54520994.38 | 139177211.7 | 1.352 | 8.27E-10 | 7.017 | TG(16:0_14:0_18:1) | TG | C51 H100 O6 N1 | 14.799 | Up |
| 4 | POS1250 | 39071530.72 | 123949088.9 | 1.666 | 4.29E-10 | 6.996 | TG(16:0_14:0_18:2) | TG | C51 H98 O6 N1 | 14.523 | Up |
| 5 | POS1419 | 66792628.03 | 151071290.1 | 1.177 | 8.17E-09 | 6.970 | TG(16:1_18:1_18:1) | TG | C55 H104 O6 N1 | 14.845 | Up |
| 6 | POS1343 | 31403358.4 | 114627514.3 | 1.868 | 9.14E-10 | 6.919 | TG(16:0_17:0_18:1) | TG | C54 H106 O6 N1 | 15.532 | Up |
| 7 | POS1248 | 36932771.88 | 112053991.9 | 1.601 | 5.83E-08 | 6.532 | TG(16:0_14:1_18:1) | TG | C51 H98 O6 N1 | 14.057 | Up |
| 8 | POS1270 | 12728757.44 | 84322878.04 | 2.728 | 3.37E-05 | 6.201 | TG(15:0_16:0_18:1) | TG | C52 H102 O6 N1 | 15.003 | Up |
| 9 | POS1459 | 36403437.94 | 99641580.9 | 1.453 | 1.72E-08 | 6.001 | TG(17:0_18:1_18:1) | TG | C56 H108 O6 N1 | 15.522 | Up |
| 10 | POS1491 | 39762683.84 | 95965266.3 | 1.271 | 1.55E-06 | 5.641 | TG(18:0_18:0_18:1) | TG | C57 H112 O6 N1 | 16.221 | Up |
| 11 | POS1379 | 89484104.07 | 144334045.3 | 0.690 | 2.32E-06 | 5.556 | TG(18:0_16:0_18:1) | TG | C55 H108 O6 N1 | 15.840 | Up |
| 12 | POS1202 | 17270975.33 | 65502421.91 | 1.923 | 2.45E-10 | 5.270 | TG(16:0_14:0_16:1) | TG | C49 H96 O6 N1 | 14.068 | Up |
| 13 | POS1351 | 32151680.44 | 81703911.04 | 1.346 | 1.92E-06 | 5.241 | TG(16:0_17:1_18:1) | TG | C54 H104 O6 N1 | 15.082 | Up |
| 14 | POS1301 | 72642244.7 | 113373115.3 | 0.642 | 1.58E-06 | 4.801 | TG(16:0_16:0_18:1) | TG | C53 H104 O6 N1 | 15.381 | Up |
| 15 | POS1425 | 16540418.94 | 51803822.93 | 1.647 | 7.86E-11 | 4.515 | TG(16:0_18:1_18:3) | TG | C55 H102 O6 N1 | 14.261 | Up |
| 16 | NEG115 | 14301720.2 | 5164958.13 | -1.469 | 1.21E-06 | 6.998 | Hex1Cer(d36:1+O) | Hex1Cer | C43 H82 O11 N1 | 8.280 | Down |
| 17 | NEG197 | 10535591.69 | 4003116.88 | -1.396 | 2.80E-12 | 6.067 | LPE(18:0)-H | LPE | C23 H47 O7 N1 P1 | 2.134 | Down |
| 18 | NEG289 | 59186.71303 | 6105490.47 | 6.689 | 0.003076124 | 5.201 | PC(16:0_18:2) | PC | C43 H81 O10 N1 P1 | 7.678 | Up |
| 19 | NEG134 | 13916871.7 | 8808294.42 | -0.660 | 0.005305332 | 4.632 | Hex1Cer(t36:1) | Hex1Cer | C43 H82 O11 N1 | 8.316 | Down |
| 20 | NEG292 | 14122366.44 | 10439360.5 | -0.436 | 1.04E-07 | 4.534 | PC(16:1e_18:1) | PC | C43 H83 O9 N1 P1 | 8.490 | Down |
| 21 | NEG587 | 3056657.193 | 6686454.92 | 1.129 | 7.83E-09 | 4.490 | PI(18:0_20:4)-H | PI | C47 H82 O13 N0 P1 | 7.129 | Up |
| 22 | NEG294 | 13054684.3 | 9642915.2 | -0.437 | 1.40E-07 | 4.352 | PC(16:1e_18:1) | PC | C43 H83 O9 N1 P1 | 8.932 | Down |
| 23 | NEG477 | 1795146.305 | 5388928.27 | 1.586 | 1.14E-05 | 4.338 | PE(36:5e)-H | PE | C41 H73 O7 N1 P1 | 8.244 | Up |
| 24 | NEG466 | 8900146.231 | 5841941.4 | -0.607 | 1.42E-09 | 4.136 | PE(18:1e_18:1)-H | PE | C41 H79 O7 N1 P1 | 10.227 | Down |
| 25 | NEG469 | 11905406.66 | 9005956.05 | -0.403 | 0.000371045 | 3.789 | PE(18:1e_18:2)-H | PE | C41 H77 O7 N1 P1 | 9.262 | Down |
| 26 | NEG573 | 1874605.286 | 4329119.03 | 1.207 | 7.03E-11 | 3.709 | PI(18:0_18:1)-H | PI | C45 H84 O13 N0 P1 | 8.268 | Up |
| 27 | NEG285 | 4802108.688 | 2537572.58 | -0.920 | 7.79E-06 | 3.482 | PC(16:0e_18:1) | PC | C43 H85 O9 N1 P1 | 8.847 | Down |
| 28 | NEG721 | 3673939.834 | 658598.218 | -2.480 | 0.041192708 | 3.220 | SPHP(m20:1) | SPHP | C21 H43 O6 N1 P1 | 2.091 | Down |
| 29 | NEG338 | 4786106.023 | 3108410.27 | -0.623 | 9.57E-09 | 3.062 | PC(18:1e_18:1) | PC | C45 H87 O9 N1 P1 | 9.780 | Down |
| 30 | NEG497 | 10084422.83 | 8409538.68 | -0.262 | 9.82E-09 | 3.048 | PE(18:0_20:4)-H | PE | C43 H77 O8 N1 P1 | 8.451 | Down |

Note: A total of 30 key lipid metabolites were selected based on the screening conditions of VIP≥1 and *P*<0.05.

**Supplemental Table 8**

Correlation analysis of 30 key lipid compounds and 6 characteristic volatile compounds

| No. | Items | Acetic acid | | Undecane | | Acrylonitrile | | Tetrahydro furane | | Ethyl acetate | | 1-Penten-3-one | |
| --- | --- | --- | --- | --- | --- | --- | --- | --- | --- | --- | --- | --- | --- |
|  |  | ρ^1)^ | *P* Value^2)^ | ρ | *P* Value | ρ | *P* Value | ρ | *P* Value | ρ | *P* Value | ρ | *P* Value |
| 1 | SPHP(m20:1) | 0.843** | 0.002 | 0.749* | 0.013 | 0.549 | 0.100 | -0.745* | 0.013 | -0.715* | 0.020 | -0.596 | 0.069 |
| 2 | Hex1Cer(t36:1) | 0.750* | 0.012 | 0.751* | 0.012 | 0.226 | 0.530 | -0.776** | 0.008 | -0.756* | 0.011 | -0.540 | 0.107 |
| 3 | PC(18:1e_18:1) | 0.962** | 0.000 | 0.976** | 0.000 | 0.699* | 0.024 | -0.982** | 0.000 | -0.990** | 0.000 | -0.854** | 0.002 |
| 4 | PC(16:1e_18:1) | 0.940** | 0.000 | 0.953** | 0.000 | 0.640* | 0.046 | -0.972** | 0.000 | -0.974** | 0.000 | -0.798** | 0.006 |
| 5 | PE(18:1e_18:1)-H | 0.952** | 0.000 | 0.971** | 0.000 | 0.687* | 0.028 | -0.980** | 0.000 | -0.986** | 0.000 | -0.798** | 0.006 |
| 6 | LPE(18:0)-H | 0.954** | 0.000 | 0.985** | 0.000 | 0.696* | 0.025 | -0.989** | 0.000 | -0.996** | 0.000 | -0.822** | 0.004 |
| 7 | PE(18:0_20:4)-H | 0.941** | 0.000 | 0.975** | 0.000 | 0.665* | 0.036 | -0.974** | 0.000 | -0.982** | 0.000 | -0.818** | 0.004 |
| 8 | PC(16:0e_18:1) | 0.941** | 0.000 | 0.975** | 0.000 | 0.665* | 0.036 | -0.974** | 0.000 | -0.982** | 0.000 | -0.818** | 0.004 |
| 9 | Hex1Cer(d36:1+O) | 0.887** | 0.001 | 0.937** | 0.000 | 0.674* | 0.033 | -0.936** | 0.000 | -0.952** | 0.000 | -0.774** | 0.009 |
| 10 | PC(16:1e_18:1) | 0.941** | 0.000 | 0.951** | 0.000 | 0.688* | 0.028 | -0.965** | 0.000 | -0.973** | 0.000 | -0.843** | 0.002 |
| 11 | TG(16:0_18:1_18:1) | 0.879** | 0.001 | 0.961** | 0.000 | 0.628 | 0.052 | -0.950** | 0.000 | -0.957** | 0.000 | -0.715* | 0.020 |
| 12 | PE(18:1e_18:2)-H | 0.777** | 0.008 | 0.815** | 0.004 | 0.634* | 0.049 | -0.834** | 0.003 | -0.864** | 0.001 | -0.869** | 0.001 |
| 13 | TG(16:0_18:1_18:3) | -0.952** | 0.000 | -0.992** | 0.000 | -0.705* | 0.023 | 0.990** | 0.000 | 0.997** | 0.000 | 0.814** | 0.004 |
| 14 | TG(16:0_17:1_18:1) | -0.941** | 0.000 | -0.966** | 0.000 | -0.688* | 0.028 | 0.968** | 0.000 | 0.973** | 0.000 | 0.787** | 0.007 |
| 15 | PI(18:0_18:1)-H | -0.955** | 0.000 | -0.996** | 0.000 | -0.647* | 0.043 | 0.993** | 0.000 | 0.994** | 0.000 | 0.805** | 0.005 |
| 16 | TG(16:0_14:1_18:1) | -0.964** | 0.000 | -0.990** | 0.000 | -0.665* | 0.036 | 0.991** | 0.000 | 0.992** | 0.000 | 0.803** | 0.005 |
| 17 | TG(16:0_14:0_16:1) | -0.958** | 0.000 | -0.990** | 0.000 | -0.665* | 0.036 | 0.988** | 0.000 | 0.995** | 0.000 | 0.836** | 0.003 |
| 18 | TG(16:0_14:0_18:2) | -0.952** | 0.000 | -0.990** | 0.000 | -0.663* | 0.037 | 0.987** | 0.000 | 0.995** | 0.000 | 0.829** | 0.003 |
| 19 | PI(18:0_20:4)-H | -0.939** | 0.000 | -0.985** | 0.000 | -0.651* | 0.041 | 0.975** | 0.000 | 0.981** | 0.000 | 0.815** | 0.004 |
| 20 | TG(17:0_18:1_18:1) | -0.945** | 0.000 | -0.988** | 0.000 | -0.671* | 0.034 | 0.988** | 0.000 | 0.989** | 0.000 | 0.793** | 0.006 |
| 21 | TG(16:0_17:0_18:1) | -0.953** | 0.000 | -0.992** | 0.000 | -0.668* | 0.035 | 0.991** | 0.000 | 0.991** | 0.000 | 0.798** | 0.006 |
| 22 | TG(16:1_18:1_18:1) | -0.952** | 0.000 | -0.988** | 0.000 | -0.676* | 0.032 | 0.990** | 0.000 | 0.992** | 0.000 | 0.782** | 0.007 |
| 23 | TG(16:0_14:0_18:1) | -0.954** | 0.000 | -0.995** | 0.000 | -0.645* | 0.044 | 0.994** | 0.000 | 0.991** | 0.000 | 0.763* | 0.010 |
| 24 | TG(18:0_17:0_18:1) | -0.951** | 0.000 | -0.985** | 0.000 | -0.655* | 0.040 | 0.989** | 0.000 | 0.987** | 0.000 | 0.758* | 0.011 |
| 25 | TG(18:0_16:0_18:1) | -0.923** | 0.000 | -0.946** | 0.000 | -0.656* | 0.040 | 0.960** | 0.000 | 0.957** | 0.000 | 0.718* | 0.019 |
| 26 | TG(16:0_16:0_18:1) | -0.936** | 0.000 | -0.963** | 0.000 | -0.517 | 0.126 | 0.968** | 0.000 | 0.955** | 0.000 | 0.745* | 0.013 |
| 27 | TG(18:0_18:0_18:1) | -0.934** | 0.000 | -0.972** | 0.000 | -0.536 | 0.110 | 0.972** | 0.000 | 0.964** | 0.000 | 0.724* | 0.018 |
| 28 | TG(15:0_16:0_18:1) | -0.867** | 0.001 | -0.929** | 0.000 | -0.561 | 0.092 | 0.924** | 0.000 | 0.942** | 0.000 | 0.816** | 0.004 |
| 29 | PE(36:5e)-H | -0.852** | 0.002 | -0.897** | 0.000 | -0.865** | 0.001 | 0.890** | 0.001 | 0.917** | 0.000 | 0.787** | 0.007 |
| 30 | PC(16:0_18:2) | -0.677* | 0.032 | -0.740* | 0.014 | -0.349 | 0.323 | 0.715* | 0.020 | 0.733* | 0.016 | 0.733* | 0.016 |

Note: ^1)^ ρ, Spearman’s rank correlation coefficient analysis. ^2)^ ** The data were considered significantly different at a *P* value < 0.01, * The data were considered significantly different at a *P* value < 0.05.
